# Synergistic Effect of Sn and Fe in Fe–N_*x*_ Site Formation and Activity in Fe–N–C Catalyst for ORR

**DOI:** 10.1021/acsami.2c13837

**Published:** 2022-12-05

**Authors:** Marco Mazzucato, Luca Gavioli, Vincenzo Balzano, Enrico Berretti, Gian Andrea Rizzi, Denis Badocco, Paolo Pastore, Andrea Zitolo, Christian Durante

**Affiliations:** † Department of Chemical Sciences, University of Padova, Via Marzolo 1, 35131Padova, Italy; ‡ i-LAMP & Department of Mathematics and Physics, Università Cattolica del Sacro Cuore, Via della Garzetta 46, 25133Brescia, Italy; § Institute of Chemistry of Organometallic Compounds (ICCOM)—National Research Council (CNR), Via Madonna del Piano 10, 50019Sesto Fiorentino, Italy; ∥ Synchrotron SOLEIL, L’Orme des Merisiers, BP 48 Saint Aubin, 91192Gif-sur-Yvette, France

**Keywords:** ORR, Fe−N−C, Sn−N−C, GDE, PEMFC, EXAFS, AEMFC

## Abstract

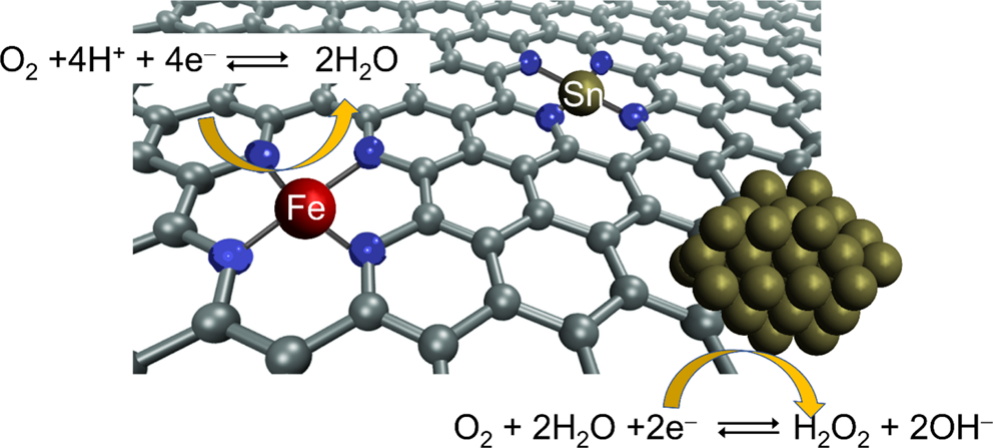

Iron–nitrogen–carbon (Fe–N–C) materials emerged as one of the best non-platinum group material (non-PGM) alternatives to Pt/C catalysts for the electrochemical reduction of O_2_ in fuel cells. Co-doping with a secondary metal center is a possible choice to further enhance the activity toward oxygen reduction reaction (ORR). Here, classical Fe–N–C materials were co-doped with Sn as a secondary metal center. Sn–N–C according to the literature shows excellent activity, in particular in the fuel cell setup; here, the same catalyst shows a non-negligible activity in 0.5 M H_2_SO_4_ electrolyte but not as high as expected, meaning the different and uncertain nature of active sites. On the other hand, in mixed Fe, Sn–N–C catalysts, the presence of Sn improves the catalytic activity that is linked to a higher Fe–N_4_ site density, whereas the possible synergistic interaction of Fe–N_4_ and Sn–N_*x*_ found no confirmation. The presence of Fe–N_4_ and Sn–N_*x*_ was thoroughly determined by extended X-ray absorption fine structure and NO stripping technique; furthermore, besides the typical voltammetric technique, the catalytic activity of Fe–N–C catalyst was determined and also compared with that of the gas diffusion electrode (GDE), which allows a fast and reliable screening for possible implementation in a full cell. This paper therefore explores the effect of Sn on the formation, activity, and selectivity of Fe–N–C catalysts in both acid and alkaline media by tuning the Sn/Fe ratio in the synthetic procedure, with the ratio 1/2 showing the best activity, even higher than that of the iron-only containing sample (*j*_k_ = 2.11 vs 1.83 A g^–1^). Pt-free materials are also tested for ORR in GDE setup in both performance and durability tests.

## 1 Introduction

Hydrogen-related technologies represent a crucial field for moving to a low-carbon economy, which is expected to offer promising opportunities not only to fight climate change but also to enhance energy delocalization and safety, to revolutionize the transport sector both for goods and people and to develop local industries in many countries.^1,2^ A clear evidence of this interest is proven by the enormous investments made in both Europe and in the United States for the development of hydrogen-based technologies, including fuel cells.^3−6^ The high cost of these devices is due to the slow kinetics of the oxygen reduction reaction (ORR) at the cathode side, and so the use of Pt-based materials as catalysts is still required.^7−9^ With their low cost, high availability, and good tolerance to poisoning, non-PGM is the best known alternative to Pt.^10,11^ During past decades, various non-PGM catalysts were investigated: M–N–C based on porphyrin-like M–N_*x*_ sites, non-precious metal oxide, chalcogenides, oxynitrides, and carbon oxynitrides.^12^ Among others, the most interesting and active are M–N–C with Fe metal center, where iron could coordinate from two up to five nitrogen functional groups,^13^ with the metal porphyrin-like Fe–N_4_ site considered as the most important for the selectivity and activity in ORR.^14,15^ A good catalyst is the result of a combination of several aspects like the site density, the intrinsic activity of sites, the carbon support hierarchical structure, the surface chemistry, the graphitization degree, and so forth.^16−20^ Choosing the right carbon matrix is the turning point to improve catalytic performance. Indeed, increasing the density of the active sites is not sufficient to enhance the catalyst activity since it is also necessary to rationally design the textural and porous properties of the carbon support to facilitate the mass transport between micropores and the bulk solution.^19,21,22^ Moreover, the catalytic enhancement can be obtained by the incorporation of heteroatoms like N, S, P, or B or another metal to form a bimetallic system.^23,24^

Bimetallic N-doped carbon has been less studied due to the already complex nature of the monometallic system. Despite this, the addition of a second metal, generally to Fe–N–C catalysts, could have an impact of site formation,^25^ final activity,^26^ or stability.^27^ For example, the addition of Ni to the precursor mixture results in a lower number of sites due to competition mechanisms during the pyrolysis,^25^ while the addition of Mn shows an activity improvement in the alkaline environment,^26^ and the use of copper seems to be beneficial to activity.^26^ Recently, Zr was used to improve the activity of Fe–N–C as well. In this case, the effect was induced by a synergistic effect between Fe–N_*x*_ site and ZrO_*x*_ present on the carbon surface.^28^

*d*-Group metals (fourth period) are generally the only used to fabricate M–N–C catalysts because of their better performances. Indeed, other metals are less interesting due to the inability to form nitrogen-coordinating single-site materials (in pyrolytic conditions) or to the tendency of forming nanoparticles (NPs) instead of isolated metal-nitrogen sites.^29,30^ Other metals are too pricy or are more prone to catalyze other reactions, such as the homogeneous CO_2_ reduction.^31−35^ In a recent paper, Strasser et al. showed the possible role of Sn in oxygen reduction reaction (ORR) in a real fuel cell system.^27^ Sn is a *p*-block element that was substantially^36^ never studied in the single-site catalyst shape, conversely to other transition metals such as Fe or Co.^37,38^ In fact, Sn is typically used as a catalyst in NP form.^39,40^ For this reason, the present work aims to study the effect of Sn on an already consolidated material made from iron phenanthroline and carbon black support^41^ to see how its addition impacts the physico-chemical and catalytical properties of the resulting material.

At the same time, it is important to develop and improve methodologies to test the catalysts under realistic conditions, which is one of the problems of rotating (ring) disk electrode (R(R)DE) analysis. Indeed, rotating techniques do not always reflect the performance on membrane electrode assembly; floating electrode and gas diffusion electrode (GDE) cells are two of the possible alternatives, whose results can be directly compared to flowing tests, leveling the problem of scaling promising materials as characterized by RRDE but showing sloppy performance in full device tests.^42−45^ As a result of this, the measured mass activity with GDE electrode technique will be a more reliable screening metric to down select materials for scale-up. Therefore, here, Pt-free materials are tested for ORR in GDE electrode setup in both performance and durability tests. Different Sn/Fe catalysts supported on Vulcan XC72 were synthesized in order to understand the role of Sn in combination with Fe. The catalytic performances were tested under acidic and alkaline conditions by means of RRDE analysis, and the site density (SD) and turn over frequency (TOF) were determined by the nitrite stripping technique.^46,47^ The most promising catalysts were also tested on a GDE setup, hoping to show the benefit of this setup, which also allowed to perform accelerated stress test (AST) in almost-like operative conditions.

## 2 Experimental Section

### 2.1 Chemicals

Iron(II) chloride (>98%), tin(II) chloride dihydrate, Nafion (5 wt % in a mixture of lower aliphatic alcohols and water), diethyl ether (>99.8%), ethanol (HPLC grade > 99.8%), THF (HPLC grade > 99.9%), acetone (HPLC grade > 99.9%), acetic acid (>99.8%), methanol (HPLC grade > 99.9%), potassium hydroxide (assay 86.7%), HCl (37%), sodium acetate trihydrate, and sodium nitrite were purchased from Sigma-Aldrich and Carlo Erba reagents and used without any purification. Vulcan XC72 was purchased from Fuel Cell Store (USA), and 1,10-phenanthroline (>99%) was purchased from Alfa-Aesar. Highly pure H_2_SO_4_ (Fluka, 93–98%, TraceSELECT) was employed for electrochemical characterization. Carbon paper with PTFE treatment and Nafion 117 membrane were purchased from Hydro2Power SRL.

### 2.2 Synthesis of Fe(phen)_3_Cl_2_ (Fe-phen) Complex

**Figure 1:**
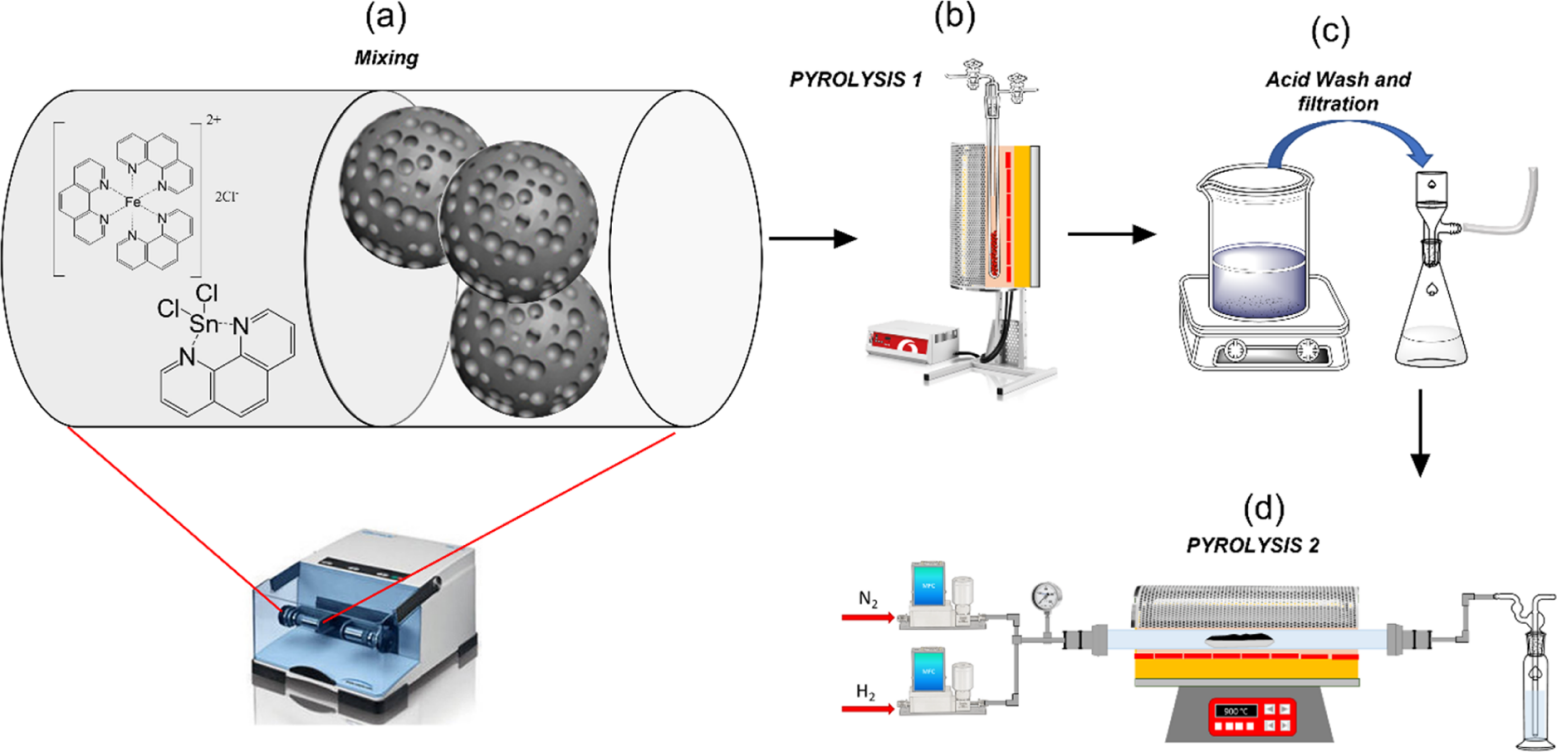
Fe-phen and Sn-phen complex structures and synthetic procedures of the Fe and Sn catalysts; (a) ball milling mixing, (b) pyrolysis step at 900 °C, (c) acid washing and filtration of the catalysts, and (d) second step pyrolysis.

Anhydrous Fe^II^Cl_2_ and 1,10-phenantroline were dissolved in a small volume of EtOH (to facilitate the further precipitation, we use the lowest necessary amount to completely dissolve the precursor) with a 1:3.1 molar ratio. The mixture was kept under stirring for 1 h, and then the complex was precipitated using diethyl ether and finally washed with diethyl ether to give a yield of about 70%^48^ (elemental analysis: C_calc_, 54.5%; N_calc_, 10.6%; H_calc_, 4.8%; C_found_, 53.9%; N_found_, 10.3%; H_found_, 4.2%). The structure is shown in Figure 1a.

### 2.3 Synthesis of Sn(phen)Cl_2_ (Sn-phen) Complex

A proper synthesis procedure was not available in the literature using 1,10-phenantroline, so it was adopted a similar procedure as for Fe(phen)_3_Cl_2_. The starting Sn compound was Sn^II^Cl_2_·2H_2_O, so the oxidation state of Sn in the complexes is +2 as compared to iron-phenanthroline synthesis. Therefore, SnCl_2_·2H_2_O and 1,10-phenantroline (1:2 molar ratio in EtOH solution) were mixed. A yellow precipitate was immediately formed; the solution was filtered within 5–10 min to avoid the decomposition product to form as observed by Owens, which adopted N oxide-phenanthroline as a ligand.^49^ The product was finally washed with ice-cold ethanol and then dried overnight at 40 °C. In alternative, the same synthesis also works in isopropanol. The yield was 92%, and the product was characterized by elemental analysis assuming that the final formula of the compound is Sn(phen)Cl_2_ as reported by Owens et al.^49^ The found elemental analysis percentages seemed to confirm that assumption (C_calc_, 38.96%; N_calc_, 7.58%; H_calc_, 2.16%; C_found_, 36.83%; N_found_, 6.82%; H_found_, 2.09%). The suggested structure is shown in Figure 1a.

### 2.4 Synthesis of Sn_*x*_Fe_*y*_–N–C Materials

The two complexes were used to synthesize of Fe–N–C catalysts as follows: 200 mg of Vulcan XC72, EC300j, MC or CBCO_2_-5 (CB Super P treated at 950 °C in CO_2_ gas flow^19^) and a suitable ratio of Sn-phen/Fe-phen complexes (different molar percentages of the metal compared to the molar amount of carbon support were chosen) were vibro-milled at 18, 20, and 25 Hz for a total of 1 h (20 min for each frequency in ascending order) (Figure 1a). Then the mixture was heated at 900 °C in a tubular furnace (Carbolite, with a quartz tube ϕ = 25 mm) for 2 h under a reductive nitrogen–hydrogen atmosphere (9% H_2_ in the mixture, HG 2400 Claind) and eventually cooled down to ambient temperature under pure nitrogen flow (Figure 1b). Fe(phen)_3_Cl_2_ was chosen as a precursor since it gives the best activity among several Fe/N complexes.^41^ The resulting powder was vibro-milled and then washed at reflux in 100 mL of 1 M solution of H_2_SO_4_ for 3 h at 100 °C under continuous stirring (Figure 1c). The suspension was then filtered and rinsed with at least 0.5 L of Milli-Q water and finally dried in oven at 40 °C overnight. Three reference samples prepared by utilizing only Sn-phen were prepared and washed, for the reason later explained (see also the Supporting Information, paragraph S1), also in 4 M HCl, 2 M H_2_SO_4_, or 2 M HNO_3_ at 90 °C for 6 h in an Ar atmosphere according to Luo et al., Supporting Information.^27^ After the acid leaching, the powder was vibro-milled or pounded in a mortar and then heated again at 900 °C as described before (Figure 1d). The resulting powders are labeled as Sn_*x*_Fe_*y*_C (where *x* and *y* are the initial percentages of tin and iron, respectively, and *C* is the name of carbon support). The catalysts were further vibro-milled before electrochemical test with the same settings listed above.

A control metal-free catalyst was synthetized adopting the same procedure but using only phenanthroline as nitrogen precursor but reaching the same molar amount as it would be with 2%_mol_ of Fe(phen)_3_Cl_2_. This sample is named as N–C along the text.

### 2.5 Electrochemical Test

Cyclic voltammetry (CV) and linear sweep voltammetry (LSV) were carried out on a rotating ring-disc electrode (RRDE, Metrohm: ϕ = 5 mm GC disk and a Pt ring for acid measurements and RRDE, Pine Instrument: ϕ = 5.61 mm GC disk and a Pt ring for alkaline measurements) in both Ar-purged and O_2_-saturated 0.5 M H_2_SO_4_ and 0.1 M KOH solution using an Autolab model 101N potentiostat or a PARSTAT 3000A-DX. All the measurements were done in a three-electrode cell thermostated at 25 °C. The RRDE tip was used as the working electrode, a graphite rod was used as the counter electrode, and for the acidic electrolyte, a homemade RHE as the reference electrode was prepared before each experiment according to the literature procedure.^14^

For the measurements in KOH, a Hg/HgO (AMEL instruments for electrochemistry) reference electrode (*E*_RHE_ = 0.098 V + 0.059pH + *E*_Hg/HgO_^0^) was adopted. The calibration of Hg/HgO reference electrode was performed in a standard three-electrode system where two polished Pt wires were the working and counter electrodes, respectively, and the Hg/HgO electrode is the reference electrode (see the Supporting Information of ref (41)).

The materials activity was investigated on a thin catalyst layer loaded on GC surface via drop-casting after the preparation of an ink made approximately of a 9:1 mixture of water, an organic solvent (acetone and THF), and Nafion (*m*_cat_/*m*_nafion solution_ ≈ 1). To obtain a good dispersion, the ink was sonicated in a bath sonicator for at least 1 h. The loading was chosen to be 0.6 mg cm^–2^ as used in previous work, knowing that it could have an impact on hydrogen peroxide detection.^19,41,50,51^

All the materials were initially activated in the Ar-purged electrolyte with extensive CV cycling at 200 mV s^–1^ until a stable current was observed. This generally lasts from 30 to 100 cycles depending on the material. The voltammetries in O_2_ free electrolyte were recorded in static and hydrodynamic conditions, and they were used to perform background subtraction of the O_2_ Faradaic current.

In ORR tests, O_2_ was bubbled inside the electrolyte solution for at least 20 min. CV at 5 mV s^–1^ and LSV at 2 mV s^–1^ 1600 rpm were recorded and then used as references to compare the activity of different materials. The number of transferred electrons (*n*) was determined from RRDE experiments according to the following equation (eq 1)
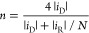
1With the RRDE analysis, it is also possible to evaluate the percentage of hydrogen peroxide (eq 2) produced at the working electrode by rearranging eq 1
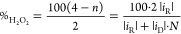
2where *i*_D_ is the current recorded at the disk, *i*_R_ is the current recorded at the ring, and *N* is the collection efficiency, which is equal to 0.25 (Metrohm, 5 mm), 0.37 (Pine, 5.61 mm), or 0.439 (Pine, 5 mm), as previously determined via analysis of Fe^2+^/Fe^3+^ redox reaction of K_4_[Fe^II^(CN)_6_].^19^ Other parameters of interest are the peak potential (*E*_p_) derived from the CV recorded in the oxygen-saturated electrolyte at 5 mV s^–1^, the half-wave potential (*E*_1/2_), and the limiting current density (*j*_lim_) determined from LSV analysis at 2 mV s^–1^ and 1600 rpm. The mass-transport-corrected kinetic current density at a selected potential was calculated according to eq 3
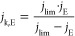
3where *j*_E_ is the current density at the selected potential *E =* 0.8 V versus RHE, but in general, *j*_k_ can be calculated at every potential in the kinetic controlled region (*j*_k,E_).

A gas diffusion electrode cell design by Arenz et al.^43^ was used to evaluate the activity in a more realistic condition since in this apparatus, the catalysts are casted on a carbon paper and covered with a Nafion membrane. The electrolyte serves as a source of proton and is not in direct contact with the catalyst layer, while the oxygen gas comes from under the layer mimicking the fuel cell cathode.

For the GDE measurement, around 4 mg cm^–2^ (we verify that loading between 3.5 and 4.5 did not result in significative change in recorded activity) was deposited on a carbon paper.

The electrolyte container was filled with 0.5 M H_2_SO_4_ as for the RRDE measurements. Graphite serves as the counter electrode and RHE as reference; the working electrode is the catalyst-loaded carbon paper supported by the steel body of the cell. The scheme of GDE cell is reported in Figure S1, and, as is possible to observe, a carbon felt layer was included between the carbon paper layer and the cell body to improve the contact between the cell and the membrane, namely, to increase the thickness and therefore the pressure when the clamp is closed.

To evaluate the catalyst site density, nitrite (NO_2_^–^) poisoning and electrochemical stripping were performed following the procedure described by Malko et al.^19,46,52^ This procedure allows the selective poisoning of Fe–N_*x*_ sites (even if adsorption on FeO_*x*_ or other sites is also possible and not easily discriminable^53^), and the site density is determined by measuring the reductive stripping charge of the adsorbed species (nitrosyl = NO) during a CV measurement. The NO is indeed formed during the poisoning step by putting the electrode in a NO_2_^–^ solution and then at acid pH. The site density measurements were performed on a thin layer of catalyst deposited on a GC (RDE, PINE Research ϕ = 5 mm) in a 0.5 M acetate buffer at pH 5.2. A loading of 0.2 mg cm^–2^ was chosen according to the published procedure.^52^ The ink was let dry with the electrode in rotation at 130 rpm for about 20 min. These measurements were carried out on a PARSTAT-3000A-DX instrument equipped with a linear CV scansion module which allows a better sensitivity of stripping charge compared to staircase voltammetry. Also, measurements with integration of the current module are a valid choice in the absence of the abovementioned one.

Stability tests were performed using an extensive cycling between 0.55 and 1.05 V as reported in the literature,^54^ which is a variation on the procedure reported by Ohma et al.^55^ for the start and stop stress tests for Pt/C fuel cell catalysts and extended also to M–N–C.^27^ The measurements were performed in the O_2_-saturated electrolyte (0.5 M H_2_SO_4_ or 0.1 M KOH) at a scan rate of 200 mV s^–1^. The activity was checked every 1000 cycles performing LSV at 5 mV s^–1^ using RRDE to evaluate also peroxide yield variation. Also, stress tests on GDE setup were carried out under chronoamperometry at 0.5 V versus RHE in both media.

### 2.6 Physico-Chemical Characterization

N_2_ adsorption/desorption isotherms were recorded at 77.3 K using an ASAP 2020 Plus instrument. The specific surface area of the samples was determined by BET analysis and with quenched solid density functional theory model, which showed to be more accurate compared to nonlinear density functional theory, even if limited to a pore dimension of 40 nm. In fact, it takes into account the roughness of the surface and the chemical heterogeneity, leading to a better fit of experimental data, in particular for disordered carbons.^56−58^ The total volume of the pore was obtained applying Gurvitsch law at *p*/*p*^0^ ≈ 0.98. Elemental analysis (EA) was carried out using a Thermo Scientific Flash 2000 analyzer.

The nano- and micro-scale morphologies of the materials were studied by scanning electron microscopy (SEM). Images were acquired with a Zeiss Sigma HD FE-SEM equipped with an INCAx-act PentaFET Precision spectrometer (Oxford Instruments) using primary beam acceleration voltages between 10 and 20 kV. The EDX elemental concentration was evaluated in three different regions for each catalyst and reported as average values in the corresponding figure.

XRD measurements were performed with a Bruker AXS D8 ADVANCE Plus diffractometer with a Cu source (λ_Kα1_ = 1.5406 Å) from 20 to 80° (2θ) with a 0.04° step on a Si zero-background sample holder (see S2 for further details).

Fe K-edge X-ray absorption spectroscopy (XAS) spectra were measured at the SAMBA beamline (Synchrotron SOLEIL) at room temperature in the fluorescence mode using a Ge 33-pixel detector. The beamline is equipped with a sagittally focusing Si 220 monochromator and two Pd-coated mirrors that were used to remove X-ray harmonics. The catalysts were pelletized as disks of 10 mm diameter using boron nitride as a binder.

The film composition was evaluated by X-ray photoelectron spectroscopy (XPS) using a Mg X-ray source (the incident photon energy was 1253.6 eV) and a Phoibos 100 SPECS analyzer at 20 eV pass energy. HR-TEM and scanning–transmission electron microscopy (STEM) EDX characterizations were performed using a TALOS F200X G2 microscope, equipped with a Super-X 4 segments EDX detector, using a beam energy of 200 keV. Compositional maps were acquired using a resolution of 512 × 512 points and a dwell time of 1 micro-s for about 20 min. Samples were prepared by a three-step procedure: (1) dispersion of a small amount of the powder in about 0.5 mL of isopropanol, (2) sonication for about 20 min, and (3) drop-casting of a drop of the dispersion onto a TEM grid.

## 3 Results and Discussion

### 3.1 Carbon Support Selection and Catalyst Preparation

**Figure 2:**
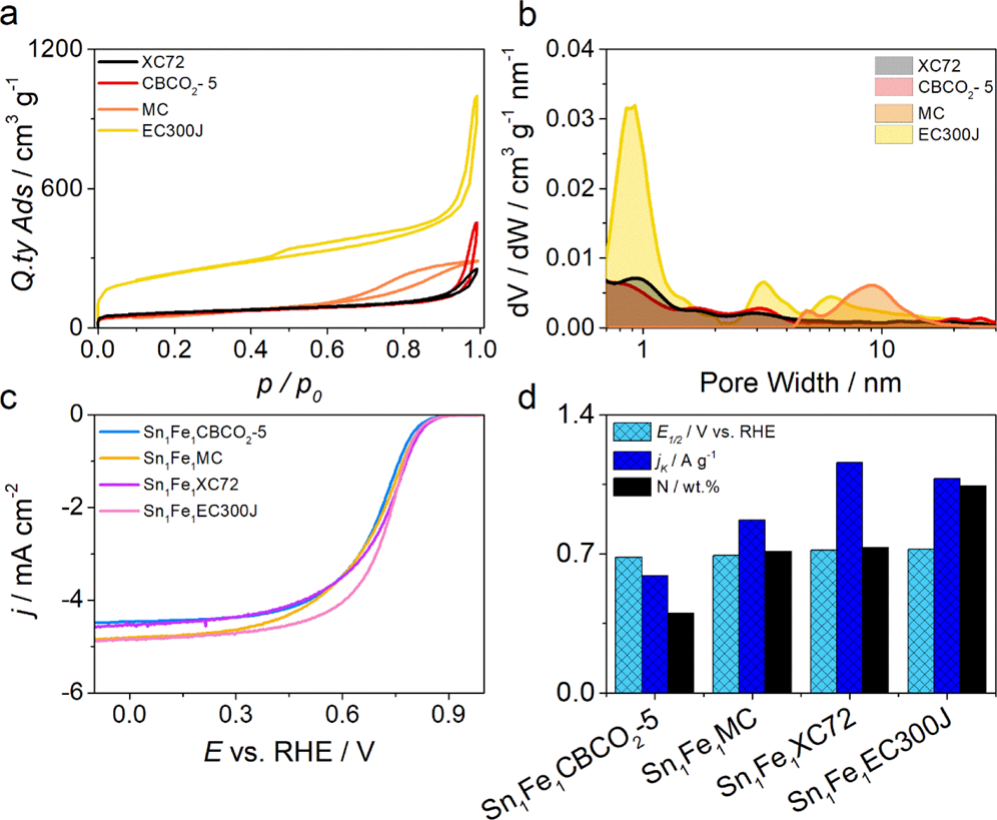
(a) N_2_ adsorption/desorption isotherms and (b) pore size distribution for the four carbon supports. (c) LSV at RDE of Sn_1_Fe_1_X recorded at 1600 rpm and 2 mV s^–1^ in O_2_-saturated 0.5 M H_2_SO_4_ and (d) summary of N content ad activity in terms of half-wave potential and kinetic current for the four Sn_1_Fe_1_X.

The first step was to select a proper carbon support for evaluating the Sn effect on the Fe–N_*x*_ site formation and activity. In this regard, four different carbons were considered, that is, Vulcan XC72, EC-300J, MC, and CBCO_2_-5, which are all commercially available with the exception of CBCO_2_-5. The latter is a commercial carbon (CB, Super P) activated with CO_2_ at 950 °C for 5 h in a tubular furnace.^19^ These four supports were chosen for the different pore structures: medium-low microporous/mesoporous carbon (CBCO_2_-5 and Vulcan XC72), highly porous carbon (EC-300J), and highly mesoporous carbon (MC), as shown in Figure 2a,b. Initially, four catalysts with equal molar contents of Sn and Fe complexes in the synthetic mixture (Sn_1_Fe_1_) but with different carbon supports were synthetized. The 1/1 composition was chosen as a preliminary composition test. The four supports were characterized by EA to detect the nitrogen content and the possible traces of sulfur (which could have an impact on the final activity^59^). N_2_ physisorption analysis was also performed for guiding the selection of the four carbon supports, among several others, on the base of the surface area and of pore size distribution that are generally not made explicit from the commercial seller (Table S1). The final choice of the carbon support was made by evaluating the one that once functionalized with Sn-phen and Fe-phen showed the best compromise between nitrogen content, according to EA, and ORR activity in terms of *E*_1/2_ and *j*_k_ determined by RRDE measurements at 1600 rpm (2 mV s^–1^) in O_2_-saturated 0.5 M H_2_SO_4_. According to the *E*_1/2_ and *j*_k_ (at 0.8 V vs RHE) parameters reported in Table S2 and Figure 2c,d, Sn_1_Fe_1_XC72 was the catalyst with better catalytic performance (*E*_1/2_ = 0.719 V vs RHE, *j*_k_ = 1.16 A g^–1^), even if similar performances were achieved with EC300J, which represent therefore a good alternative (*E*_1/2_ = 0.722 V vs RHE, *j*_k_ = 1.08 A g^–1^). On the basis of these findings, Vulcan XC72 was selected as a support to carry out all the other Sn/Fe combinations. Specifically, five different catalysts with different Sn-phen/Fe-phen ratios were prepared (Sn_*x*_Fe_*y*_XC72), along with two samples with only Sn (Sn_*x*_XC72 with *x* = 2, 4) and one sample with only iron (Fe_2_XC72).^41^

### 3.2 Physico-Chemical Characterization of Sn_*x*_Fe_*y*_XC72 Catalysts

**Figure 3:**
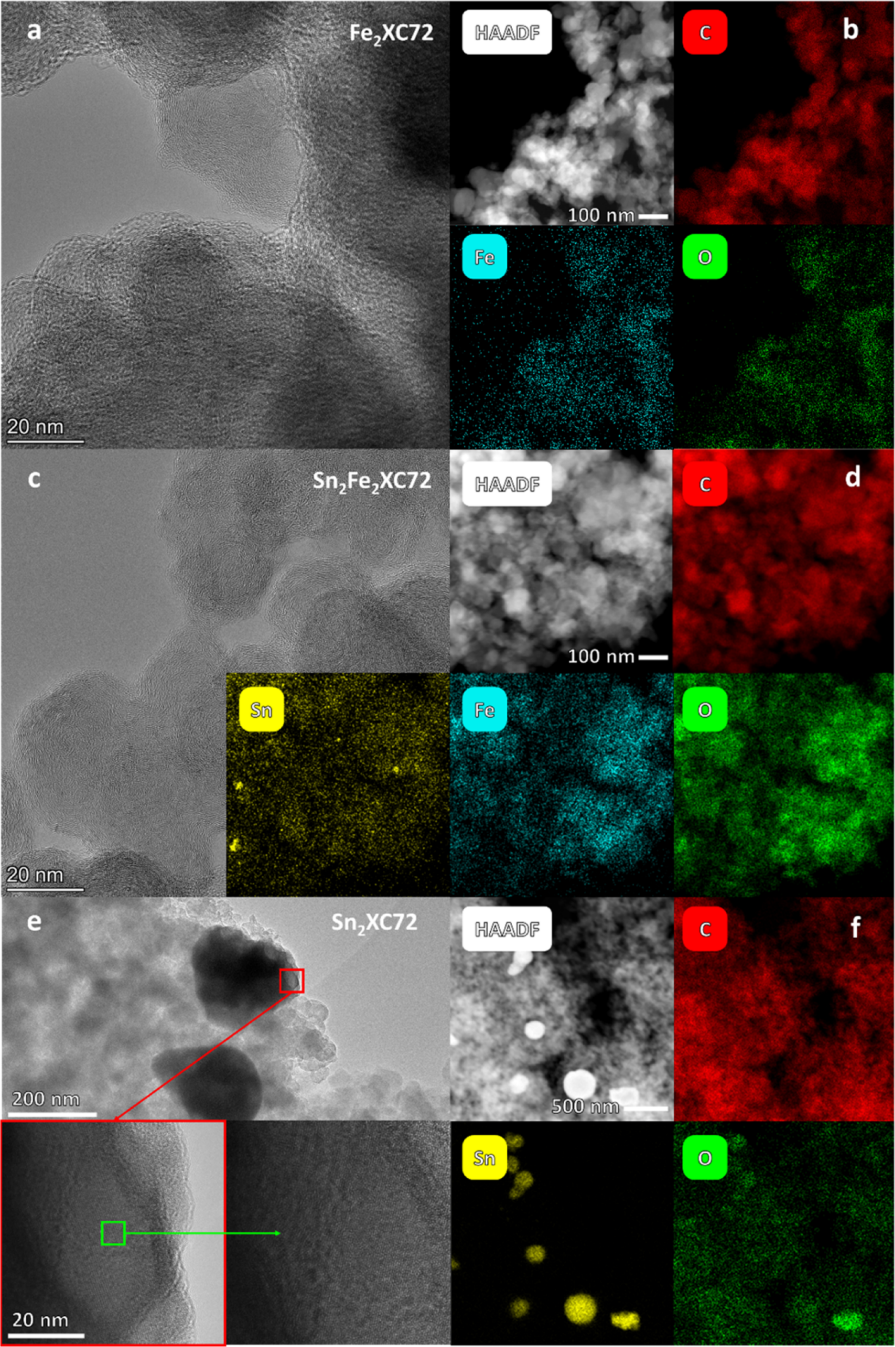
HR-TEM and HAADF-STEM for Fe_2_XC72, Sn_2_Fe_2_XC72, and Sn_2_XC72. (a) TEM and (b) HAADF-STEM with elemental mapping for Fe_2_XC72; (c) TEM and (d) HAADF-STEM with elemental mapping for Sn_2_Fe_2_XC72; (e) TEM and (f) HAADF-STEM with elemental mapping for Sn_2_XC72.

The morphological/compositional aspects of Sn_*x*_Fe_*y*_XC72 catalysts were defined by HR-TEM (Figure 3) and SEM–EDX analysis (Figure S2), EA, and ICP–MS (Table 1). The dispersion of Fe and Sn on the catalysts was evaluated by high-angle annular dark field–STEM (HAADF-STEM) coupled with elemental mapping (Figure 3). For Fe_2_XC72 (Figure 3a,b), an exploration of the sample indicates the absence of metal particles and a general structure similar to that of a carbon powder. STEM EDX underlines the presence of Fe and O homogeneously distributed on the carbon and compatible with the single-site nature of the catalyst. The exploration of Sn_2_Fe_2_XC72 (Figure 3c,d) gives a structure compatible with that of Fe_2_XC72, with the sporadic presence of metallic tin NPs, of the dimensional order of 20–50 nm. The STEM EDX acquisitions performed on C showed a clear Fe peak, which appears to be homogeneously distributed in the sample, consistent with the “atomically dispersed”, Sn signal that was not very evident, but elemental mapping shows a dispersion that could be compatible with a Sn single-atom dispersion. Sn_2_XC72 (Figure 3e,f) shows a dispersion of NPs, mostly of a spherical shape. The smeared shape of Sn NPs is indicative of the melting of NPs upon the thermal treatment. From the STEM EDX analysis, the particles appear to be composed of metallic Sn. The acquisitions performed out of the Sn NP do not show peaks relative to Sn; however, the presence of atomically dispersed Sn, which could be present in very low concentrations, cannot be ruled out.

**Table 1:** Composition of the Catalysts Derived from EA (C, N, H, S), EDX (C, O, S, Fe, Sn), and ICP–MS (Fe, Sn)

	C_EA_(wt %)	N_EA_(wt %)	H_EA_(wt %)	S_EA_(wt %)	C_ED_*x*__(wt %)	O_ED_*x*__(wt %)	S_ED_*x*__(wt %)	Fe_ED_*x*__(wt %)	Sn_ED_*x*__(wt %)	Fe_ICP_(wt %)	Sn_ICP_(wt %)
Sn_1_Fe_1_XC72	91.03	0.73	0.32	0.33	97.02	2.34	0.28	0.36	0.05	0.20	0.04
Sn_2_Fe_2_XC72	89.54	1.28	0.46	0.26	94.71	4.20	0.24	0.69	0.17	0.45	0.11
Sn_2_Fe_1_XC72	95.43	0.87	0.24		96.98	2.47	0.02	0.39	0.15	0.27	0.12
Sn_3_Fe_1_XC72	93.51	1.44	0.26	0.26	96.06	3.12	0.21	0.46	0.15	0.37	0.09
Sn_1_Fe_2_XC72	92.87	0.94	0.34	0.16	96.99	2.34	0.17	0.51	0.00	0.29	0.04
Sn_4_XC72	85.94	0.83	0.30	0.23	85.20	2.77	0.20		11.83		7.20
Sn_2_XC72	92.16	0.27	0.18		93.13	1.45			5.43		4.60
Fe_2_XC72	90.68	0.82	0.40	0.32	95.72	3.24	0.40	0.64		0.34	
N–C	98.55	0.23	0.17								

Samples were further characterized by SEM, and samples with Fe or Fe and Sn show only agglomerations of carbon NPs as will be further confirmed by XPS and XRD analysis, even if different brightnesses could induce to different conclusions (Figure S2). The carbon NPs have dimensions in the order of 100 nm, in line with literature findings on XC72 carbon.^60,61^ It is confirmed that when only Sn-phen is used as a metal precursor, a dispersion of bright Sn NPs spanning from 100 to 1000 nm form (see later XRD analysis). Sn NPs can be regarded as metallic Sn NPs enveloped in SnO_*x*_, which is reasonable considering the reductive atmosphere (high temperature and hydrogen gas) and the ex situ exposition to air. The metallic nature was already confirmed by the fact that elemental mapping on Sn_2_XC72 does not show always a higher concentration of O in correspondence of Sn NPs (Figure 3f). This result justifies the lower carbon content recorded by elemental analysis (Table 1) for the two samples Sn_2_XC72 and Sn_4_XC72 where Sn wt % over 4% was detected (Table 1). This result might suggest two things: (i) the acid leaching is not effective in removing Sn NPs possibly formed during the first pyrolysis step (or alternatively during the second pyrolysis step, during which an insoluble salt can form during the acidic treatment), and/or (ii) the presence of Fe does not allow Sn NPs to form or in a more general way hinders the fixation of Sn in the matrix. This last hypothesis would find confirmation by both ICP–MS and SEM–EDX that in the case of Sn give wt % lower than 0.2% in Sn_*x*_Fe_*y*_XC72 catalysts. It is interesting to note that the synthesis temperature (900 °C) is over the melting point of tin; therefore, the carbon morphology helps to form NP dispersion instead of bigger agglomerates since tin should be in liquid form at this temperature.

The formation of Sn NPs only in certain conditions prompted us to study more in depth the effect of the acid leaching on Sn_4_XC72 by employing different acid solutions, that is, HCl, HNO_3_, and more concentrated H_2_SO_4_ (see S1 in the Supporting Information for nomenclature and Table S3). It was observed that nitric acid is quite ineffective since the percentage of tin in the Sn_4_N2T sample remains high (4.89%). HCl brings some improvements in removing Sn NPs in Sn_4_C2T (1.17%), but H_2_SO_4_ actually remains the best choice, even if the conditions, 3 h at 100 °C in 1 M H_2_SO_4_, successfully adopted for Fe_*x*_XC72 are not sufficiently effective for Sn_*x*_XC72. Probably the main issue was the high Sn content and a not enough time of treatment, coupled with a slow reaction in a diluted acid. In fact, only doubling the H_2_SO_4_ concentration as well as the washing duration (6 h at 90 °C under N_2_ in 2 M H_2_SO_4_ similarly to Luo et al.^27^) allows to lower the Sn concentration (0.90%) of 1 order of magnitude, and the NP dispersion becomes less evident (Figure S2). The electrochemical behavior of Sn_4_XC72 after the different acid washing was tested in both O_2_ saturated 0.5 M H_2_SO_4_ and 0.1 M KOH (Figure S3). A more in-depth discussion on this point can be found in the Supporting Information. It is however worth to say that the catalytic performances remain similar for the Sn_*x*_XC72 treated in different acidic conditions (Tables S4 and S5); perhaps, some improvements can be claimed especially for the sample treated in nitric acid, but overall, the activities are lower with Fe_2_XC72 sample taken as a reference. Similar findings were observed in an alkaline environment, where the selectivity versus H_2_O_2_ increases to almost 50% (Figure S3). This seems to suggest that in acidic conditions, Sn NPs have not a central role in ORR activity. The same is not true in an alkaline environment, where at least at the negative potential (below 0.3 V vs RHE), a secondary mechanism where H_2_O_2_ is reduced to H_2_O seems to be present. Indeed, for a higher amount of Sn NPs, the amount of peroxide is lower, suggesting some role of Sn in ORR catalysis (Figure S3c–e). Even if not the central point of this work, the study of Sn NPs for ORR was not found in the literature, which is why it was retained worth to note here.

The nature of NPs was further investigated by means of XRD analysis that shows that only metallic Sn phases are evident, while no clear peaks from SnO or SnO_2_ were detected (see XRD analysis, S2 and Figure S4). This is reasonable considering the reductive atmosphere during synthesis, but we cannot exclude that a low-crystalline layer of oxide is present on the surface since EDX, as said, sometimes detected oxygen when NPs are analyzed (Figure 3f).

If we now look at the metal composition derived from ICP and EDX in Sn_*x*_Fe_*y*_XC72 catalysts (Table 1), it can be observed that iron concentration scales in good agreement with the amount of phenanthroline added in the precursor mixture with the two complexes (Fe-phen and Sn-phen), suggesting that the additional phenanthroline from Sn-phen helps fixing both nitrogen and iron. This also well correlates with SD that will be discussed later in the text. This leads to the conclusion that it does not matter how phenanthroline is added in the mixture since an increment in iron and Fe–N_*x*_ is observed (Figure S5) regardless of whether the nitrogen source is Fe-phen, Sn-phen, or additional phenanthroline (see also S4 in the Supporting Information; in particular, the sample marked as 1* in Figure S11). This is understandable since the amount of Fe initially added is at least 1 order of magnitude higher than the final amount in the catalysts; in other words, it is more important of being able to fix iron instead of having it available in the initial precursor mixture. In any case, we already saw that the 2 molar percentage is optimal for this type of synthesis.^41^ This does not belittle the role of Sn-phen, in particular to obtain Sn–N_*x*_ formation, but we want to stress out the role of phenanthroline to achieve Fe–N_4_ fixation.

XPS performed on selected samples (Fe_2_XC72, Sn_1_Fe_2_XC72, Sn_2_Fe_2_XC72, and Sn_4_XC72) shows the signals of N, Fe, and Sn. All samples show a characteristic N 1s peak with multiple components assignable to pyridinic, pyrrolic, graphitic, and M–N_*x*_ (Figure S6). For Sn_4_XC72, the component relative to M–N_*x*_ could be linked to Sn–N_*x*_. Fe 2p deconvolution shows the presence of Fe^2+^ and Fe^3+^, which is in line with the finding for Fe–N–C material.^19^ Finally, Sn 3d peak shows that Sn is present as Sn^4+^ in all samples, in line with the finding of Luo et al.;^27^ for Sn_4_XC72, this shows that, as expectable, the surface of metallic NPs is oxidized but not in the crystalline form. The small shoulder at the peak foot (Figure S6i) could be linked to the metallic tin signal. Quantitative analysis was not elaborated since the amount is close to the detection limit.

**Figure 4:**
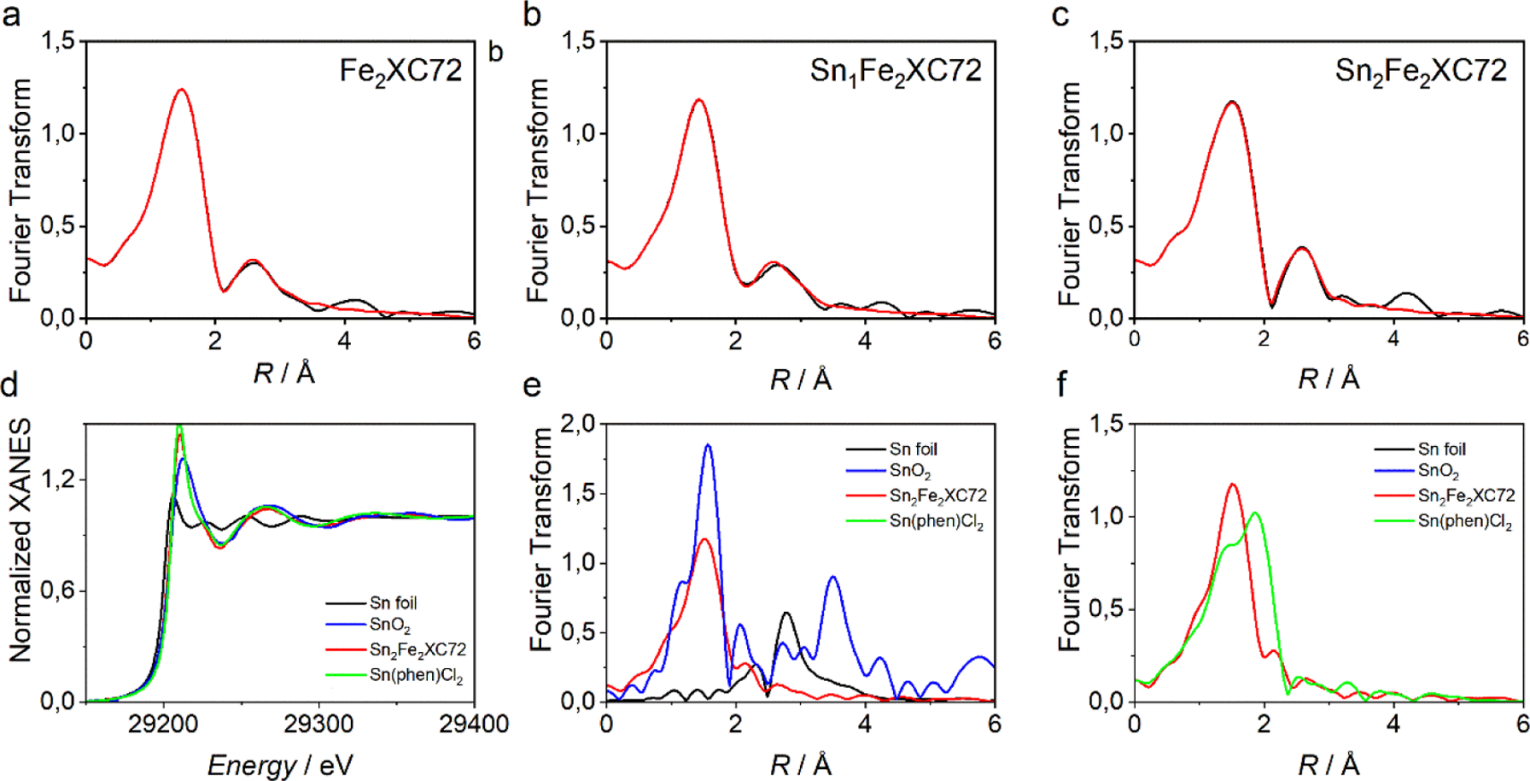
Fe K-edge EXAFS analysis in the Fourier transformed space of (a) Fe_2_XC72, (b) Sn_1_Fe_2_XC72, and (c) Sn_2_Fe_2_XC72. The black curves represent the experimental spectra, while the red curves represent the calculated spectra without phase-shift correction applied. (d) Comparison between the Sn K-edge XANES experimental spectra of Sn_2_Fe_2_XC72 (red curve), SnO_2_ (blue curve), crystalline tin (black curve), and Sn-phen complex (green curve). (e) Fourier transform of the experimental EXAFS spectra of crystalline tin (black curve), Sn_2_Fe_2_XC72 (red curve), and SnO_2_ (blue curve). (f) Fourier transform of the experimental EXAFS spectra of Sn_2_Fe_2_XC72 (red curve) compared to Sn-phen complex (green curve).

To further identify the structure of Fe–N_*x*_ moieties, XAS measurements were performed at the Fe K-edge on three selected catalysts, namely, Fe_2_XC72, Sn_1_Fe_2_XC72, and Sn_2_Fe_2_XC72. A detailed extented X-ray absorption fine structure (EXAFS) analysis shows that the Fe atoms are atomically dispersed on the carbon matrix in all the samples (see S3, Figures 4, S7, and S8), with the Fourier transform (FT) of the EXAFS signal characterized by a first peak at ca 1.5 Å assigned to the Fe–N first coordination shell and a minor peak at 2.6 Å assigned to Fe–C backscattering from the second coordination shell.^62^ These findings are fully in agreement with HAADF-STEM analysis which revealed the homogeneous dispersion of iron sites all over the catalyst. The EXAFS spectra were fitted assuming the presence of in-plane nitrogen atoms and also oxygen atoms as axial ligands (Figure S7). The structural parameters obtained from the analysis are summarized in Table S6, and they show a Fe–N coordination number around 4 for all the catalysts, demonstrating that iron forms Fe–N_4_ moieties in both the Sn-free and Sn-containing samples. Moreover, an axial signal between Fe and a light atom can be probed, suggesting that two O atoms are adsorbed on top of the Fe–N_4_ moieties, as previously found in other Fe–N–C catalysts.^63,64^ No Fe–Fe backscattering is detectable in all the EXAFS spectra, thus confirming the absence of metal NPs. As far as the X-ray absorption near-edge structure (XANES) region of the spectrum is concerned, the Fe K-edge XANES spectra of Fe_2_XC72, Sn_1_Fe_2_XC72, and Sn_2_Fe_2_XC72 are superimposed (Figure S8), further demonstrating that the same Fe-based active sites are present in all the catalysts.

To obtain additional atomic-scale information on the nature of Sn active sites, we resorted to EXAFS spectroscopy also for Sn K-edge. Figure 4d reports the Sn K-edge XANES spectra of Sn_2_Fe_2_XC72, SnO_2_, Sn-Phen complex, and crystalline tin. The threshold energy of the Sn K-edge XANES spectrum of Sn_2_Fe_2_XC72 sensitively differs from the response of SnO_2_ and metallic Sn. The Fourier transform of the Sn_2_Fe_2_XC72 EXAFS spectrum (Figure 4e) shows the dominant contribution of a first-shell peak at 1.52 Å associated with the coordination of light atoms. Contributions that would be characteristic for metallic tin and SnO_2_ are not present or are only of minor entity in Sn_2_Fe_2_XC72, in agreement with STEM EDX analysis and as is evident from the comparison of the signals of the three different species in Figure 4e. The spectrum of Sn_2_Fe_2_XC72 is partially superimposable with Sn-phen, and the main bonding distance in Sn_2_Fe_2_XC72 is also superimposable with the second major feature in Sn-phen which is also found in Sn(IV) phthalocyanines and assigned to Sn–N bond distance.^27^ The other peak could be related to Sn–Cl bond. Furthermore, the Sn K-edge XANES spectrum of Sn_2_Fe_2_XC72 is superimposable with the response of similar catalysts reported in the literature presenting Sn–N_4_ sites.^27^ Therefore, it can be concluded that Sn–N_*x*_ sites are present in Sn_2_Fe_2_XC72 and a similar consideration can be extended to the other Sn_*x*_Fe_*x*_XC72 catalysts, also supported by HR-TEM. It is worth to stress that the superimposable shape of Fe K-edge XANES spectra of Fe_2_XC72, Sn_1_Fe_2_XC72, and Sn_2_Fe_2_XC72 suggests that Fe center has the same electron density in the valence d-band so that any electronic effect induced by Sn–N_*x*_ active sites can be excluded.

### 3.3 Electrochemical Characterization

**Figure 5:**
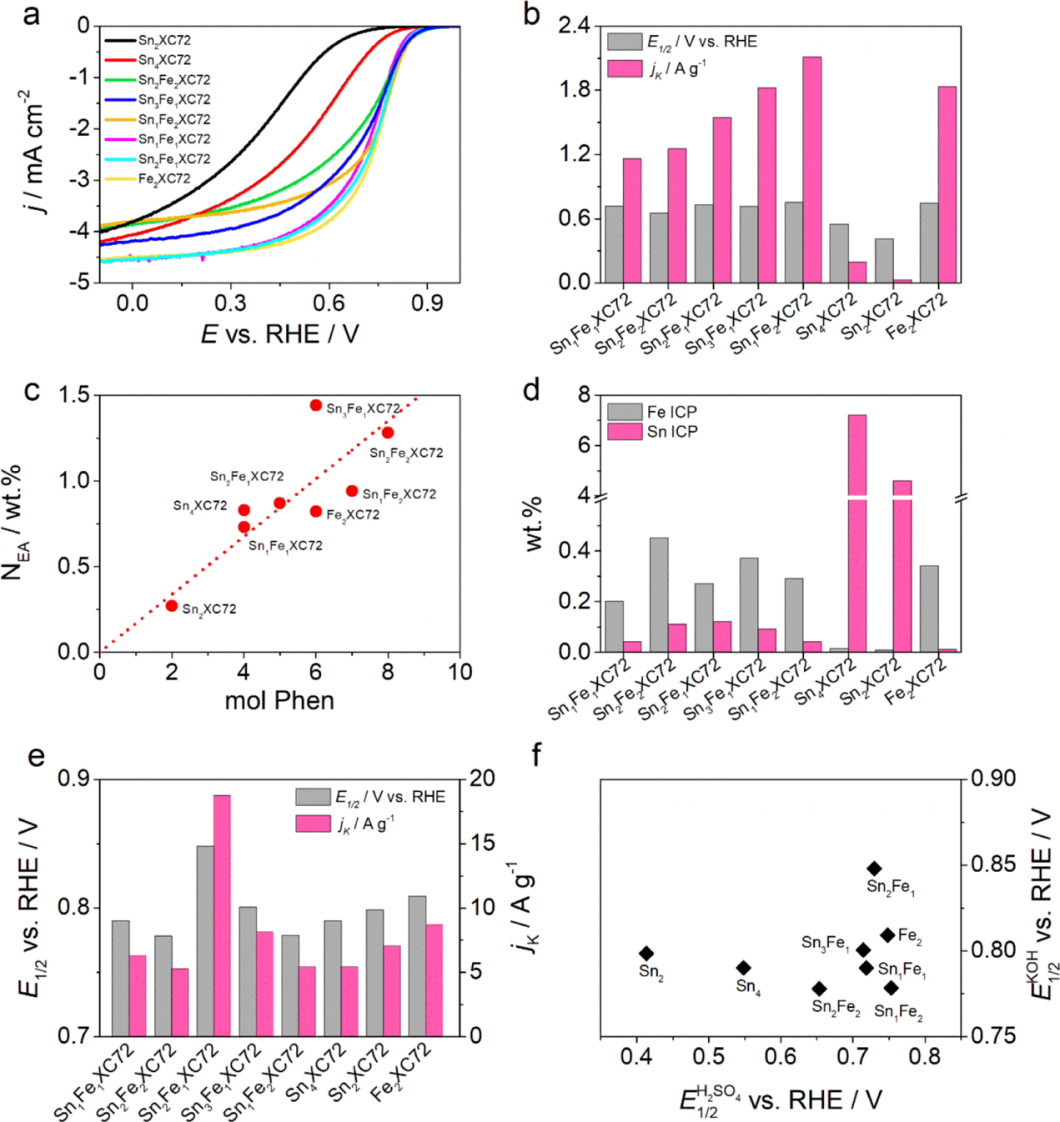
(a) LSV at RDE of investigated catalysts in O_2_-saturated 0.5 M H_2_SO_4_, (b) activity comparison in terms of half-wave potential and kinetic current for the acid electrolyte, (c) correlation between phenanthroline amount in the precursor mixture and nitrogen fixation, (d) metal content in the catalysts derived from ICP–MS, (e) activity comparison in terms of half-wave potential and kinetic current for the alkaline electrolyte, and (f) comparison of half-wave potential in the two electrolytes; the absence of correlation is clear.

**Figure 6:**
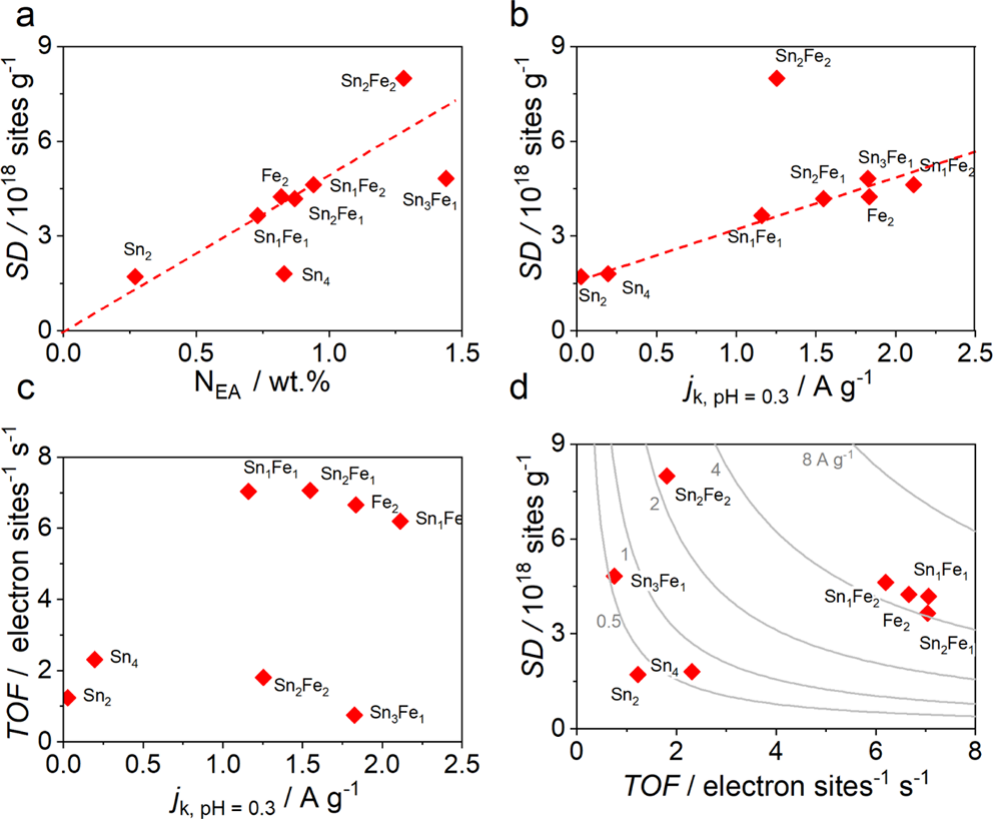
(a) Correlation of SD with N content. (b,c) SD and TOF vs an activity parameter (*j*_k_). The red dotted line is intended as a guide and not as fitting of data. (d) SD-TOF map with the iso-current curve.

All the samples were characterized in both acid and alkaline electrolytes, and the results are listed in Table 2 and Figure 5. In H_2_SO_4_, the activity for the sample containing only Sn increases when more Sn-phen is added to the initial precursor mixture. In fact, the half-wave potential increases from 0.413 to 0.548 V versus RHE and the kinetic current passes from 0.03 to 0.19 A g^–1^ (Table 2). We underline however that these two samples do not show a clear limiting current plateau (Figure 5a). Considering that in the final catalyst the amount of Sn is proportional to the precursor mixture, we can in first instance hypothesize that Sn NPs have a role in activity, but the experiments carried out on the Sn_4_X samples treated with different acids would lead to other conclusions; that is, decreasing the Sn content and so the number of Sn NPs does not lead to a decrease of activity. Another note is that if Sn NPs have an activity, this should be negligible since the weight amount of Sn is very high in these samples. As a matter of fact, the activity even seems to increase as if the removal of Sn NPs could lead to a better exposure of other active sites. The only reason for such a behavior in Sn_4_X catalysts is the formation or exposition of carbon topological defects, nitrogen defects, or Sn–N_*x*_ sites, whose presence has been confirmed by XPS, EXAFS, and HAADF-STEM characterizations. These considerations together do not allow to fully understand the main role of Sn in these catalysts; what is certain is that Sn_2_XC72 and Sn_4_XC72 performed better than a N-doped carbon (N–C, Figure S9), in particular Sn_2_XC72 that has the same amount of N (around 0.3 wt %), which could be another signal of active site differences. On the other hand, Fe_2_XC72 shows a very good activity (*E*_1/2_ = 0.748 V vs RHE and *j*_k_ = 1.83 A g^–1^), which is comparable with that of the bimetallic system (Figure 5b).^41^ The best performances are observed for Sn_1_Fe_2_XC72 (*E*_1/2_ = 0.753 V vs RHE and *j*_k_ = 2.11 A g^–1^). In this case, the larger amount of iron combined with the presence of Sn–N_*x*_ or the effect of the additional source of phenanthroline gives a better fixation/formation of active sites that, as reported later, is also in line with the nitrite stripping results (Figure 6). This statement is also in line with the growing activity passing from Sn_1_Fe_1_XC72 and Sn_2_Fe_1_XC72 to Sn_3_Fe_1_XC72 (Figure 5b). In fact, nitrogen and iron fixation (Figure 5c,d) and also SD (Figure 6) increase with the increase of the amount of Sn-phen in the initial mixture. The possibility that Sn–N_*x*_ sites also grow as the Sn-phen complex amount increases cannot be ruled out, although stronger evidence would be needed to support this hypothesis. In fact, the variation of Sn content in the different samples as determined by ICP–MS (Table 1) gives more credit to the idea that the main role in activity is due to Fe, but in any case, the addition of tin is not harmful for the overall activity/site formation, as observed for other systems with Ni.^25^ This means that the presence of Sn complex in the mixture does not compete with Fe–N_*x*_ site formation, but at most the opposite is true, that is, that Fe does not allow Sn NPs to form.

**Table 2:** Electrochemical Results for All the Catalysts in O_2_-Saturated 0.5 M H_2_SO_4_ and 0.1 M KOH

	0.5 M H_2_SO_4_				0.1 M KOH			
	*E*_1/2_^a^(V)	*j*_k_^0.8V^ (A g^–1^)	*n*_0V_	% H_2_O_2_^0.7V^	*E*_1/2_^a^(V)	*j*_k_^0.8V^ (A g^–1^)	*n*_0V_	% H_2_O_2_^0.7V^
Sn_1_Fe_1_XC72	0.719	1.16	3.98	2.2	0.790	6.31	3.15	1.0
Sn_2_Fe_2_XC72	0.654	1.25	3.98	0.8	0.778	5.26	3.06	3.1
Sn_2_Fe_1_XC72	0.729	1.55	3.98	0.8	0.848	18.75	3.42	1.6
Sn_3_Fe_1_XC72	0.715	1.82	3.95	1.7	0.801	8.13	3.25	1.5
Sn_1_Fe_2_XC72	0.753	2.11	3.97	1.0	0.779	5.43	3.18	3.3
Sn_4_XC72	0.548	0.19	3.97	2.2	0.790	5.44	3.65	4.8
Sn_2_XC72	0.413	0.03	3.97	3.0	0.799	7.05	3.70	12.5
Fe_2_XC72	0.748	1.83	3.99	0.6	0.809	8.70	3.53	1.0

a All the potentials are referred to RHE.

Passing to an alkaline electrolyte, we observe a lower difference between samples if the onset or half-wave potentials (Table 2, Figure S10) are considered as activity descriptors; Sn_2_Fe_1_XC72 (*E*_1/2_ = 0.848 V vs RHE) results to be the most active, followed by Sn_3_Fe_1_XC72 (*E*_1/2_ = 0.801 V) and Sn_1_Fe_1_XC72 (*E*_1/2_ = 0.790 V). The trend in catalytic activity apparently does not match with the nitrogen, iron, or tin content, converse of that, for example, suggested by Li et al., where a good correlation between atomically dispersed Fe atom and the onset potential was found in an alkaline electrolyte.^65^ Indeed, also Sn_4_ and Sn_2_ perform significantly well.^41^ Interestingly, only these two samples clearly present double-step LSV (Figures S3 and S10a), which corresponds with a maximum trend for the % H_2_O_2_ determined at the ring (Figures S3 and S10b). This current profile is also present for the Sn_*x*_Fe_*y*_XC72 series, even if, in this case, the second step is much less accentuated. This behavior suggests that H_2_O_2_ forms at the first step and it is then further reduced in the second step. Therefore, the presence of Sn involves a preferential 2 + 2 reduction mechanism, which is different from that observed in iron-containing ones, where H_2_O_2_ is produced, even in not a negligible amount, but it is harder to be further reduced to H_2_O at more negative potentials. In the case of Sn_*x*_XC72, the presence of NPs could have a different role, at least in an alkaline electrolyte, catalyzing the preferential formation of H_2_O_2_ and its further reduction. This is clearly seen by correlating the peroxide yield with the amount of tin detected by EDX (Figure S3e).

It can be observed that the activity is not strictly comparable between the two different electrolytes (Tables 2 and S5 and Figure 5c–f). This finding is common for this type of material, meaning that the active site switches their activity by changing the electrolyte pH. In general, the peroxide production results higher in the alkaline electrolyte (close to 50%, Table 2), suggesting a mixed 4/2 + 2/2 electron pathway different from that observed in acids where the highest yield was under 5% along the whole range of potentials analyzed (Table 2). It is known from the literature that in metal porphyrin-like systems, the Fe–N_4_ metal center is inactive when Fe is in the III redox state because of poisoning by strongly adsorbed OH^–^.^66^ Similarly, pyridinic N has higher ORR activity in an alkaline medium than in an acid one because the protonation in the acidic medium suppresses the catalytic activity.^67^ This can explain on one hand the low performances of Fe–N_4_-containing catalysts in alkaline electrolytes and the comparable performance of tin-containing catalysts. The fact that at different pH different active sites come into play is made explicit by the graph in Figure 5f, where no correlation is observed with a performance descriptor such as *E*_1/2_ in the two different electrolytes.

Stripping analysis, which allows site density determination, returns a picture in agreement with that previously observed for activity; that is, SD well correlates with the nitrogen content in the sample, the half-wave potential, and kinetic current (Figure 6a,b and Table S7), and this was already observed in previous papers.^19,41^ Before going into the merits of the question, it is worth observing the results we recorded for the stripping tests on a nitrogen-doped XC72 carbon (N–C) without metals and on Sn_*x*_XC72. N–C is expected to not give any stripping charge, and so SD equal to zero should be considered; in the true, a non-negligible SD of 1.95 × 10^17^ sites g^–1^ was found. In the case of N–C, even if it is questionable whether the active site could give NO adsorption, the found value can be regarded as the limit of detection for the techniques or simply as a sort of background (Figure S11d). The SD determined for Sn_4_XC72 is 1.71 × 10^18^ sites g^–1^, but as for N–C, we cannot be sure about the active sites that are involved in the NO_2_ adsorption and stripping (Figure S11c). Considering the large Sn content in the sample, it is clear that only a small fraction contributes to adsorb NO, even if it could also be argued that the NO adsorption on Sn is not strong or chemically reversible. In addition, Sn_4_XC72 has almost a double amount of Sn compared to Sn_2_XC72 but has almost the same SD. Considering that, we can conclude that a stripping charge from Sn NPs is unlikely or very low, whereas a contribution from Sn–N_*x*_ can be neither confirmed nor excluded. In the best catalysts (Sn_2_Fe_1_XC72, Sn_1_Fe_2_XC72, Fe_2_XC72), the increment in activity is mainly due to an increment of Fe–N_4_ SD (and/or Sn–N_*x*_) since the TOF is similar. Concerning the TOF, a clear trend with respect to N content or other kinetic parameters is not observed (Figure 6c), maybe caused by different site distributions (different sites with different activities). For example, Sn_2_Fe_2_XC72 shows high SD (Figure 6b) but at the same time a lower TOF, which results in a lower activity compared with the best catalysts (Figure 6c). In other words, despite a similar Fe–N_*x*_ local structure, as shown by XAS, some other parameters seem to influence the intrinsic activity. In fact, a high SD density could result in too close sites, which interfere with each other with the result that they are not active at the same time and in the same way. Sn_3_Fe_1_XC72 has a different combination of SD and TOF; SD correlates with activity in the acid electrolyte, while the recorded activity in acetate buffer (stripping condition) seems underestimated, which probably also affects the determination of TOF.

As anticipated, the same trend with SD is not observed in the case of an alkaline environment, suggesting the different roles of Fe species in catalyzing the reaction under alkaline conditions.^41^ Different works indeed suggested that the absence of pyridinic nitrogen protonation, the presence of Fe–N_*x*_ sites near pyridinic nitrogen, the deactivation by OH^–^ adsorption into Fe–N_4_ centers (and on Sn–N_*x*_), and the presence of FeC_*x*_ NPs under the carbon layer indicate more active sites for ORR under basic conditions.^67−69^

The conclusion of these measurements is that catalysts prepared starting from Sn/Fe precursor show better catalytic performance, where Sn-phen improves Fe fixation and the formation of Sn–N_*x*_, which however seem to not have a determinant role in affecting the catalytic activity of Fe–N_4_ (EXAFS spectra are not indicative of a different electronic structure of Fe site in the presence or absence of tin sites, and TOF does not improve). It was observed that the sole employment of Sn-phen leads to the formation of Sn NPs, which do not appear to sensitively affect the ORR in acid electrolytes but have a role in alkaline media since a clear 2 + 2 ORR mechanism was observed only for Sn_*x*_XC72 catalysts. This last finding represents in any case an interesting novelty that was not previously observed in the literature up to our knowledge.

Performing RRDE is an easy way to characterize materials to obtain information on activity and selectivity, but at the same time, it is also known that these types of characterizations fail in predicting the real behavior that a catalyst stumbles across in a real device (fuel-cell cathode). Several approaches have been proposed in order to reach closer conditions, that is, the floating electrode^45^ or the gas diffusion electrode setup as proposed here.^43^

The setup proposed by Arenz et al. allows an easy preparation of the measurements since only the drop-casting or spray-coating of catalysts on a carbon paper/gas diffusion layer is required and no hot pressing or similar procedure is needed. The contact between the layer and the membrane is indeed guaranteed by the pressure generated by the upper part of the cell clamped on the bottom part.

**Figure 7:**
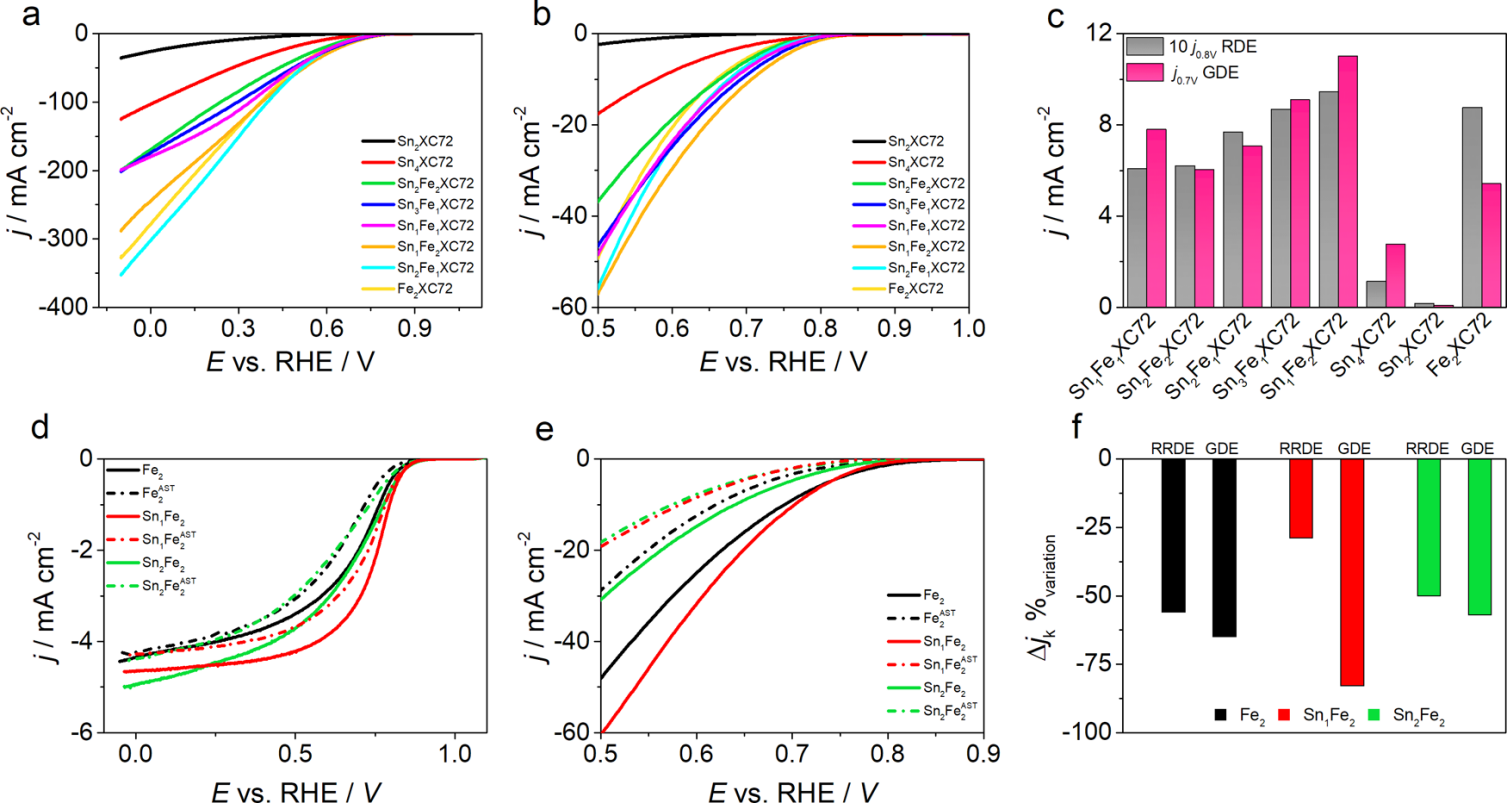
(a) Cyclic voltammograms recorded in GDE setup; only the cathodic scan is reported for clarity; (b) magnified onset region of (a); (c) current density comparison between the two setups. LSV before and after (−·−) AST in (d) RRDE and (e) GDE setup. (f) Variation (%) of current density at certain potentials for the two configurations.

All the catalysts were then tested in this setup (Figure S1), showing a kinetic growing current and the absence of a limiting current as in RRDE measurements (Figures 5c and 7a). It is evident that the scale of the current density reached by GDE setup is almost 50 times higher than in RRDE setup, at least for the most active samples (Table S8 for extended data). The activity trend in terms of kinetic current density at 0.7 V versus RHE scales in the order of Sn_1_Fe_2_XC72 > Sn_3_Fe_1_XC72 > Sn_1_Fe_1_XC72 > Sn_2_Fe_1_XC72 > Sn_2_Fe_1_XC72 ≈ Fe_2_XC72 ≫ Sn_4_XC72 > Sn_2_XC72, and it is in good agreement with RRDE measurements with the exclusion of Fe_2_XC72 (Figure 7c and Table 3). In general, Sn_*x*_Fe_*y*_XC72 catalysts at GDE perform better than Fe_2_XC72, and this can be attributed to a higher number of active Fe–N_4_ sites. It is worth to stress that samples that are a little out of trend could be influenced by a better dispersion on the carbon paper or to a higher “affinity” with this setup; in other words, they could be the candidate out of the group for device application.

**Table 3:** Results for GDE and RRDE Accelerated Stress Test in Terms of Current Variation of at Potentials Specified in the Note

	Δ*j*^a^(mA cm^–2^)	%_var_	Δ% H_2_O_2_
Fe_2_XC72^RRDE^	–0.24	–53%	–2.7
Sn_1_Fe_2_XC72^RRDE^	–0.23	–26%	–0.9
Sn_2_Fe_2_XC72^RRDE^	–0.32	–47%	–2.3
Fe_2_XC72^GDE^	–19	–40%	
Sn_1_Fe_2_XC72^GDE^	–41	–68%	
Sn_2_Fe_2_XC72^GDE^	–13	–41%	

a 0.8 V versus RHE for RDE and 0.5 V for GDE.

One of the key points of this measurement and maybe the most important one in our purpose is to show that this setup is very easily applicable to M–N–C in particular considering that, at present, only few papers adopted this method, mainly on Pt or other metals.^42,70,71^ One of possible future perspectives is to close the cycle and verify how the result obtained in GDE translates to a real fuel-cell device in order to better understand the connection between the result obtained in laboratory conditions with RDE or GDE and the actual performance under realistic conditions.

To conclude our analysis on catalyst performance, we chose to perform accelerated stress test (AST) on three different catalysts, that is, Fe_2_XC72, Sn_1_Fe_2_ XC72, and Sn_2_Fe_2_ XC72 (also characterized by XAS). ASTs are indeed fundamental since good activity and durability are two crucial points in selecting a good catalyst candidate. The selected catalysts belong to a series where the amount on tin precursor is incremented in the mixture, keeping in mind that this led to a higher concentration of Fe and N active sites rather than an actual Sn fixation. Since we perform analysis on two experimental setups (RRDE and GDE), we carried out stress tests on both. For the rotating electrode setup, we used a multiple cycling technique, while for GDE, a potentiostatic analysis was used.^41^ Results are shown and summarized in Figure 7d,f and Table 3.

Fe_2_XC72 tested at RRDE showed a higher resistance to deactivation in an alkaline environment with a loss of activity of about 10% after 7000 cycles (ca. 5 mV loss at a half-wave potential), while the activity loss in an acid electrolyte was exceptionally pronounced (ca. −60%). On the same catalysts, we also performed an AST in GDE configuration, this time comparing the activities before and after a chronoamperometry at 0.5 V versus RHE maintained for 8 h, by performing CV at 20 mV s^–1^. With this setup, the Ar background was also recorded and utilized for background subtraction. Figure 7e,f and Table 3 report the obtained data.

Sn_1_Fe_2_XC72 revealed a higher activity than the other catalysts in both setups, but the stability resulted quite different. In RRDE analysis, Sn_1_Fe_2_XC72 resulted more stable than the other two, while in GDE cell configuration, it was the reverse. On one hand, this shows that in GDE configuration, the relation between activity and stability is inversely proportional; on the other hand, this shows that not always the stability recorded on glassy carbon rotating electrode is the same as in another setup. In general, the test under potentiostatic conditions is more detrimental than multiple cycling, or simply, the 7000 cycles are not sufficiently effective as a stress test as compared to chronoamperometry in GDE conditions. In any case, with the exception of Sn_1_Fe_2_XC72, the other two (Fe_2_XC72 and Sn_2_Fe_2_XC72) show similar loss in current in both setups. This different behavior in degradation could be linked to different aspects: first, the different activity recorded is probably linked to some differences in sites. This cannot apply to the Fe–N_4_ sites because the EXAFS measurements found them to be the same for the three samples. It could therefore be related to the specific degradation of the Sn–N_*x*_ sites rather than pyridinic or pyrrolic functional groups since these should be present in different amounts as the amount of tin complex in the synthesis mix varies. Second, other textural property differences could lead to different stabilities/activities.

## 4 Conclusions

In the present paper, the evaluation of the possible cooperative effect between Sn and Fe active sites in the ORR was evaluated. Catalysts were prepared starting from a different Sn/Fe ratio of the respective complexes in the precursor mixture and supported on Vulcan XC72, which was proved to lead to better catalytic performance with respect to other carbon supports. XAS and HR-TEM measurements confirmed the formation of both Fe–N_4_ and Sn–N_*x*_ sites, and NO nitrite stripping confirmed that the site density increases in samples where Sn-phen was increasingly added, probably for the additional source of phenanthroline, which would improve the formation of Fe–N_4_ sites rather than for a synergistic effect between Fe–N_4_ and Sn–N_*x*_ active sites. In fact, the EXAFS responses for Sn_2_Fe_2_XC72 and Fe_2_XC72 are superimposable, confirming a similar electronic structure of the Fe active site. Indeed, the catalytic activity versus ORR in 0.5 M H_2_SO_4_ grows passing from Sn_1_Fe_1_XC72 and Sn_2_Fe_1_XC72 to Sn_3_Fe_1_XC72, and *E*_1/2_ and *j*_k_ scale linearly with the SD, which in turn scales with the nitrogen and iron fixation in the carbon matrix. It was observed that the sole employment of Sn-phen leads to the formation of Sn NPs, which do not appear to sensitively affect the ORR in the acid electrolyte. A correlation between SD and activity was not observed in the case of an alkaline environment, suggesting the different roles of Fe species in catalyzing the reaction under alkaline conditions. Furthermore, in an alkaline electrolyte, Sn NPs have an effect on the selectivity rather than on activity since a clear 2 + 2 ORR mechanism was observed only for Sn_*x*_XC72 catalysts.

Catalysts were also tested at GDE in acid electrolytes, and the findings are in good agreement in terms of activity with RRDE measurements with the exclusion of Fe_2_XC72. The same setup was also used for stability tests, which evidenced a far more important loss in activity than that predicted from RRDE stress tests, pointing out the importance of implementing new forms of catalyst screening for possible scale-up rather than relying on the sole rotating technique tests.

As a general remark, Sn co-functionalization does not appear to bring to tangible improvements on both catalytic activity and stability of Fe–N–C. In fact, even if a certain improvement was observed, this was appointed to the general increase of N fixation that in turn leads to an increase of Fe–N_4_ site formation.

## Supporting Information Available

Effect of different acid leaching conditions; XRD analysis; EXAFS data analysis; effect of phenanthroline; and XPS, SEM, and RDE analyses (PDF)
