# Supplementary material for: Synergistic Effect of Sn and Fe in Fe–Nx Site Formation and Activity in Fe–N–C Catalyst for ORR
Source: ACS Appl Mater Interfaces. 2022 Dec 5;14(49):54635–48. doi: 10.1021/acsami.2c13837 (PMC9756292; doi:10.1021/acsami.2c13837)
Supplement: Supplementary file 1 — am2c13837_si_001.pdf [file am2c13837_si_001.pdf]

Supporting Information

Synergistic Effect of Sn and Fe in Fe-N_x site formation and activity in Fe-N-C Catalyst for ORR

*Marco Mazzucato[1], Luca Gavioli[2], Vincenzo Balzano[2], Enrico Berretti,[3] Gian Andrea Rizzi[1], Denis Badocco[1], Paolo Pastore[1], Andrea Zitolo[4] and Christian Durante[1,]**

[1]Department of Chemical Sciences, University of Padova, via Marzolo 1, 35131, Padova, Italy.

[2]i-LAMP & Department of Mathematics and Physics, Università Cattolica del Sacro Cuore, via della Garzetta 46, 25133, Brescia, Italy

[3]Institute of Chemistry of Organometallic Compounds (ICCOM)—National Research Council (CNR), via Madonna del Piano 10, 50019 Sesto Fiorentino, Italy

[4]Synchrotron SOLEIL, L'Orme des Merisiers, BP 48 Saint Aubin, 91192 Gif-sur-Yvette, France

*christian.durante@unipd.it

Table of content

Supporting Text 2

S1 Effect of different acid leaching condition

S2 XRD Analysis

S3 EXAFS data analysis

S4 Additional investigation

Supporting Tables 5

Supporting Figures 8

Supporting References 16

Supporting Text

S1 Effect of different acid leaching condition

The presence of Sn NP in Sn_4XC72 suggested that the acid leaching (see experimental section) is not able to dissolve Sn or to convert it in a soluble salt (which remain after vacuum filtration) preventing the effectiveness of this treatment. In order to remove the NP, we try several conditions also looking at literature work where Sn was used [1]:

- HCl 4M for 6h in Ar-saturated solution at 90°C
- HNO_3 2M for 6h in Ar-saturated solution at 90°C
- H_2SO_4 2M for 6h in Ar-saturated solution at 90°C

For each sample after washing a fraction was set aside and the rest was submitted to the second heat treatment. The sample was labelled: Sn_4X (where X = acid used) after acid leaching and Sn_4W2T (where $\boldsymbol{W}$ = S for H_2SO_4, N for HNO_3 and C for HCl).

In Table S3 we report the elemental analysis for each step and in Figure S2 and Figure S3 respectively the SEM-EDX analysis and the electrochemical performances.

Sn_4C2T has a higher percentage of carbon which might suggest that higher Sn has been leached out but looking at SEM-EDX analysis appear that the sample with the highest leaching, namely with the lower evidence of NPs (Figure S3), is the sample treated in 2 M sulphuric acid (Sn_4S2T), while nitric acid is the less effective. In any case the prolonged acid treatment (6 h vs. 3 h) and the higher concentration of the acid allow for all 3 considered leaching bath formulation a better leaching compare with the Sn_4XC72.

Compared to the original Sn_4XC72 the sample leached with HNO_3 shows better performance, while Sn_4C2T and Sn_4S2T shows very similar activity with the latter that perform very similar to the other sample leached in H_2SO_4. The same is not true in alkaline electrolyte where Sn_4XC72 outperformed the other sample and also shows a different LSV profile where a clear double sigmoidal shape is present, in correspondence of the second step the amount of peroxide produced decrease where for the other sample remain almost constant (the same is also true for Sn_2XC72, see main text)

S2 XRD analysis

XRD analysis was performed on Sn_4XC72 in order to understand the nature of Sn NP, Figure S4 does not show any peak related to SnO_2 while almost all sharp peaks are associable with metallic Sn, 3 additional peaks were not related with tin species but assigned to Si, probably gives by the substrate. This means that if tin oxide is present in the outer layer of nanoparticles isn't in a crystalline form. Lastly two very broad peak at around 25 and 45° (very broad and almost superimposable with the background) are associated to the crystalline part of carbon support, as typical for M-N-C materials. Materials with Fe and Sn/Fe does not show peaks associated with inorganic crystalline phases due to confirming the effectiveness of acid leaching, remembering that, as said, in presence of iron no NPs were observed.

S3 EXAFS data analysis

The EXAFS data analysis was performed with the GNXAS code, which is based on the decomposition of the EXAFS $\chi(k)$ signal into a summation over n-body distribution functions $\gamma(n)$ calculated by means of the multiple-scattering (MS) theory. Details of the theoretical framework of the GNXAS approach are described in refs [2,3]. The Fe coordination shells have been modelled with Γ-like distribution functions which depend on four parameters, namely, the coordination number N, the average distance R, the mean-square variation σ^2, and the skewness β. Note that β is related to the third cumulant C_3 through the relation $C_3=\sigma^3\beta$.

S4 Additional investigation

The sample Sn_2XC72 and a metal free prepared with only phenanthroline [4] in the mixture were used to show that Sn_2XC72 shows a non-negligible stripping charge from NO_2 stripping analysis (Figure S10), which means that something in the sample gives a response to NO_2. We think that is unlikely that this is due to NP since the amount is very high, so we cannot exclude that some SnN_x are present in the sample.

To verify that also an effect of the sole additional phenanthroline is present a sample with 1%$_{mol}$ of initial amount of Fe was prepared and another one with an additional amount of phenanthroline to reach the same quantity added for the 2% (marked as 1*); for more detail see reference [4].

Fe_1 as expected is worse than the sample with the 2, but the sample prepared with 1% of Fe and additional phenanthroline well perform, suggesting that also a simple effect of Sn-phen complex as phenanthroline source could be present, indeed the activity of Sn_1Fe_1 was only slightly better. This at the end means that the amount of Fe in the precursor is sufficient at 1%, but additional N-source is necessary to fix more sites to reach better acidity and selectivity (comparing 1 and 1* in Figure S10).

S5 TEM Sample preparation

Samples prepared by dispersion of powders in isopropanol, sonication and subsequent dropcasting on C holey copper screen.

Supporting Tables

Table S1: N_2 Physisorption result for the four supports under analysis

	S_{tot} [a] $m^2\ g^{-1}$	$S_{0.5\text{-}40nm}$ [b] $m^2\ g^{-1}$	S_{μ} [b] $m^2\ g^{-1}$	S_{meso} [b] $m^2\ g^{-1}$	V_{μ} [b] $cm^3\ g^{-1}$	V_{meso} [b] $cm^3\ g^{-1}$	V_{tot} [c] $cm^3\ g^{-1}$
**$CBCO_2$-5**	199	197	129	68	0.055	0.457	0.791
**MC**	207	186	7	179	0.002	0.427	0.444
**XC72**	233	234	179	55	0.075	0.231	0.380
**EC300J**	806	779	423	356	0.172	0.671	1.494

[a]BET Model, [b]QSDFT Model, [c]Gurvich rule at 0.98

Table S2: Electrochemical result for the 4 samples prepared for choosing the best support

	$E_{j=0.1}$ V vs. RHE	$E_{1/2}$ V vs. RHE	E_p V vs. RHE	$j_k^{0.8V}$ A g^{-1}	n_{0V} -	$\%H_2O_2^{0.7V}$ -
**$Sn_1Fe_1CBCO_2$-5**	0.839	0.683	0.755	0.59	3.98	1.1
**Sn_1Fe_1MC**	0.850	0.692	0.756	0.87	3.97	2.3
**Sn_1Fe_1XC72**	0.857	0.719	0.771	1.16	3.98	2.2
**Sn_1Fe_1EC300J**	0.856	0.722	0.769	1.08	3.98	2.3

Table S3: Elemental analysis and Sn content (EDX) for the 3 samples washed in different acidic solutions, the elemental analysis is reported for each step while the EDX only on the final catalyst

	C_{EA} wt.%	N_{EA} wt.%	H_{EA} wt.%	Sn_{EDX} wt.%
Sn(phen)Cl_2 + XC72	63.93	3.86	1.37	-
After 1° thermal tret.	80.72	1.64	0.21	-
**Sn_4HCl**	78.97	1.40	0.38	-
**Sn_4HNO_3**	84.19	1.74	0.56	-
**$Sn_4H_2SO_4$**	86.11	1.69	0.34	-
**Sn_4C2T**	91.68	1.17	0.21	1.17
**Sn_4N2T**	83.27	0.96	0.22	4.89
**Sn_4S2T**	86.14	1.31	0.25	0.90

Table S4: Electrochemical result in 0.5 M H_2SO_4 for the 4 samples prepared from the 3 acid leaching

	$E_{i=0.1}$ V vs. RHE	$E_{1/2}$ V vs. RHE	$j_{L,0V}$ mA cm^{-2}	$j_{0.8\ V}$ mA cm^{-2}	$j_k^{0.8V}$ A g^{-1}	n_{0V} -
**Sn_4XC72**	0.803	0.548	3.72	0.11	0.19	3.97
**Sn_4C2T**	0.774	0.498	3.72	0.06	0.11	3.97
**Sn_4N2T**	0.831	0.624	4.84	0.25	0.44	3.98
**Sn_4S2T**	0.815	0.592	3.86	0.14	0.25	3.97

Table S5: Electrochemical result in 0.1 M KOH for the 4 samples prepared from the 3 acid leaching

	$E_{i=0.1}$ V vs. RHE	$E_{1/2}$ V vs. RHE	$j_{L,0V}$ mA cm^{-2}	$j_{0.8\,V}$ mA cm^{-2}	$j_k^{0.8V}$ A g^{-1}	n_{0V} -
**Sn_4XC72**	0.933	0.790	3.78	1.75	5.44	3.65
**Sn_4C2T**	0.912	0.762	4.18	1.46	3.75	3.11
**Sn_4N2T**	0.909	0.779	3.77	1.52	4.25	3.32
**Sn_4S2T**	0.916	0.766	4.16	1.58	4.26	3.10

Table S6: Best-fit parameters obtained from the EXAFS analysis of Fe_2XC72, Sn_1Fe_2XC72, and Sn_2Fe_2XC72. R (Å) is the interatomic distance, σ^2 (10^{-3} Å^2) is the Debye-Waller factor and N is the coordination number. Errors are given in parentheses, *e.g.*, 2.04(2) means 2.02-2.06

	R_{Fe-N} (Å)	σ^2 (10^{-3} Å^2)	N_{Fe-N}	R_{Fe-O} (Å)	σ^2 (10^{-3} Å^2)	N_{Fe-O}
**Fe_2XC72**	2.04(2)	8.2(4)	3.7(4)	1.89(2)	10(2)	2.0(2)
**Sn_1Fe_2XC72**	2.04(2)	12(1)	3.8(3)	1.90(2)	10(2)	2.0(3)
**Sn_2Fe_2XC72**	2.03(2)	7.0(3)	4.0(4)	1.86(2)	9.3(5)	2.0(2)

Table S7: Stripping data for the analysed samples

	Q_{STRIP} C g^{-1}	Δj_k A g^{-1}	*SD* sites g^{-1}	*MSD* mol sites g^{-1}	*TOF (0.8V)* s^{-1}
**Sn_1Fe_1XC72**	2.92	4.11	3.64E+18	6.04E-06	7.04
**Sn_2Fe_2XC72**	6.40	2.11	7.99E+18	1.33E-05	1.81
**Sn_2Fe_1XC72**	3.35	4.81	4.18E+18	6.94E-06	7.06
**Sn_3Fe_1XC72**	3.86	0.57	4.82E+18	8.01E-06	0.75
**Sn_1Fe_2XC72**	3.70	4.08	4.62E+18	7.67E-06	6.20
**Sn_4XC72**	1.44	0.66	1.80E+18	2.98E-06	2.31
**Sn_2XC72**	1.37	0.34	1.71E+18	2.84E-06	1.23
**Fe_2XC72**	3.39	4.52	4.24E+18	7.03E-06	6.66
**NC**	0.16	0.00	1.95E+17	3.23E-07	0.00

Table S8: Electrochemical result for all the catalysts in GDE cell

	$E_{i=2}$ V vs. RHE	j_{0V} mA cm^{-2}	$j_{0.7\,V}$ mA cm^{-2}
**Sn_1Fe_1XC72**	0.767	180.00	7.79
**Sn_2Fe_2XC72**	0.752	170.00	6.03
**Sn_2Fe_1XC72**	0.760	303.00	7.06
**Sn_3Fe_1XC72**	0.776	175.00	9.09
**Sn_1Fe_2XC72**	0.789	245.00	11.00
**Sn_4XC72**	0.721	103.00	2.76
**Sn_2XC72**	0.519	25.54	0.07
**Fe_2XC72**	0.748	279.00	5.42

Supporting Figure

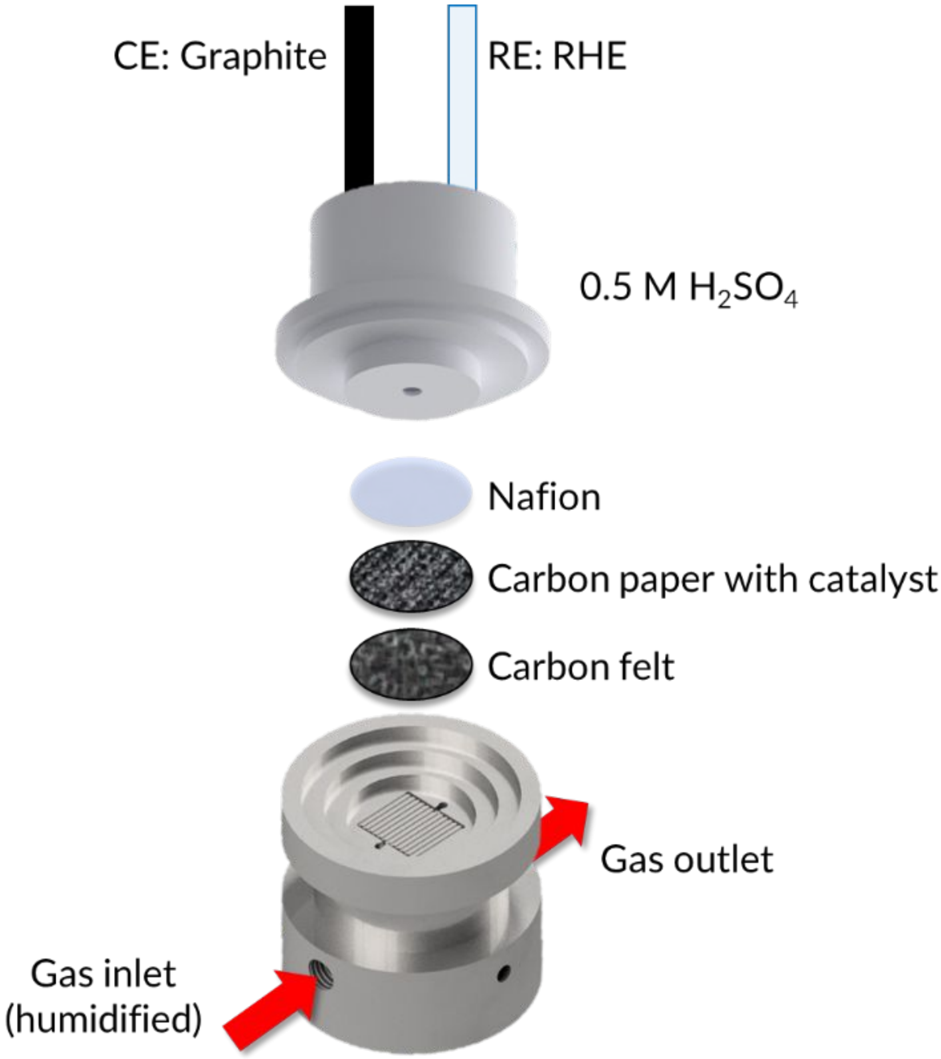

Figure S1: GDE cell set-up of our cell based on the scheme proposed by the inventor and seller [3,4]

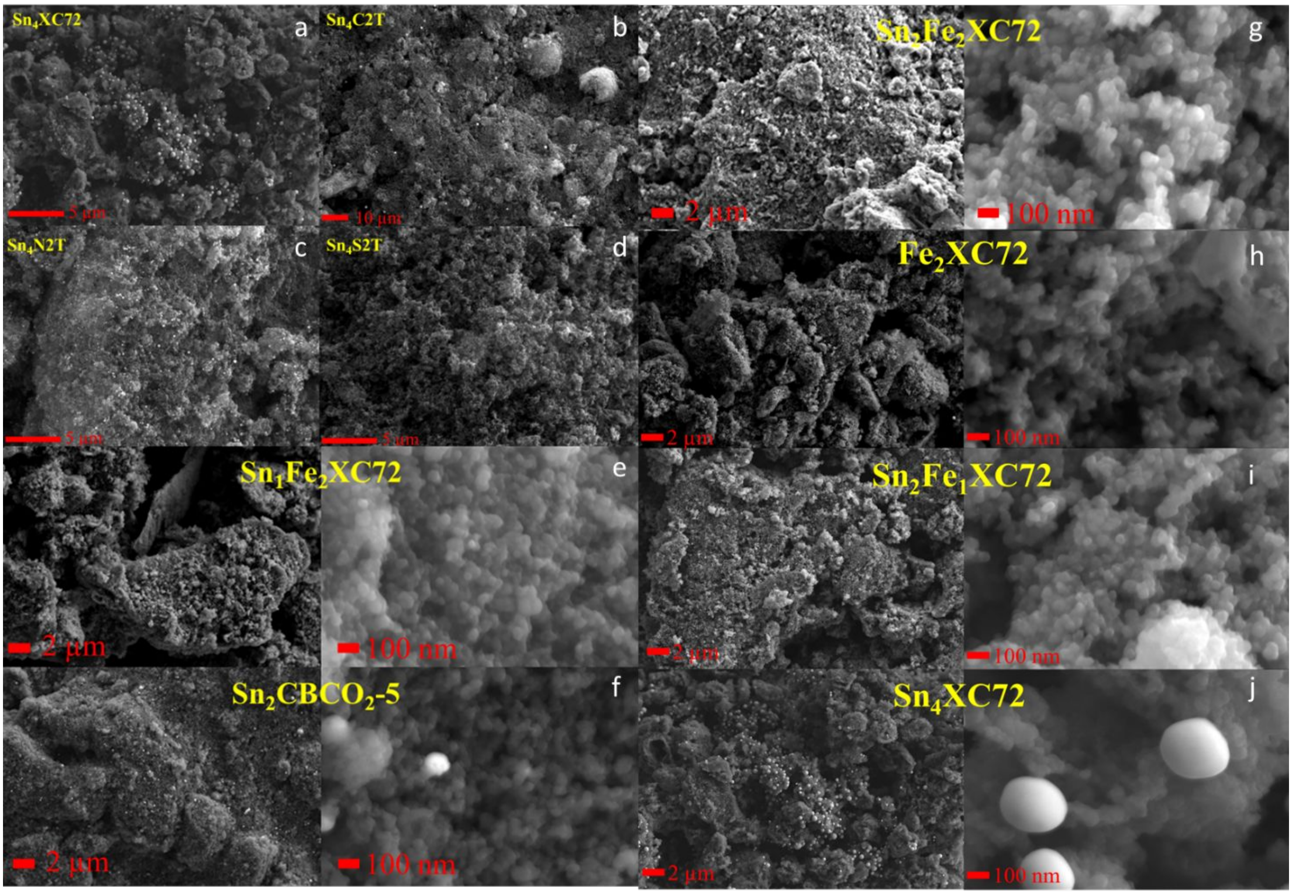

Figure S2. SEM image of a) Sn_4XC72, b) Sn_4C2T, c) Sn_4N2T, d) Sn_4S2T, e) Sn_1Fe_2XC72, f) Sn_2CBCO_2-5, g) Sn_2Fe_2XC72, h) Fe_2XC72, i) Sn_2Fe_1XC72 and j) Sn_2XC72.

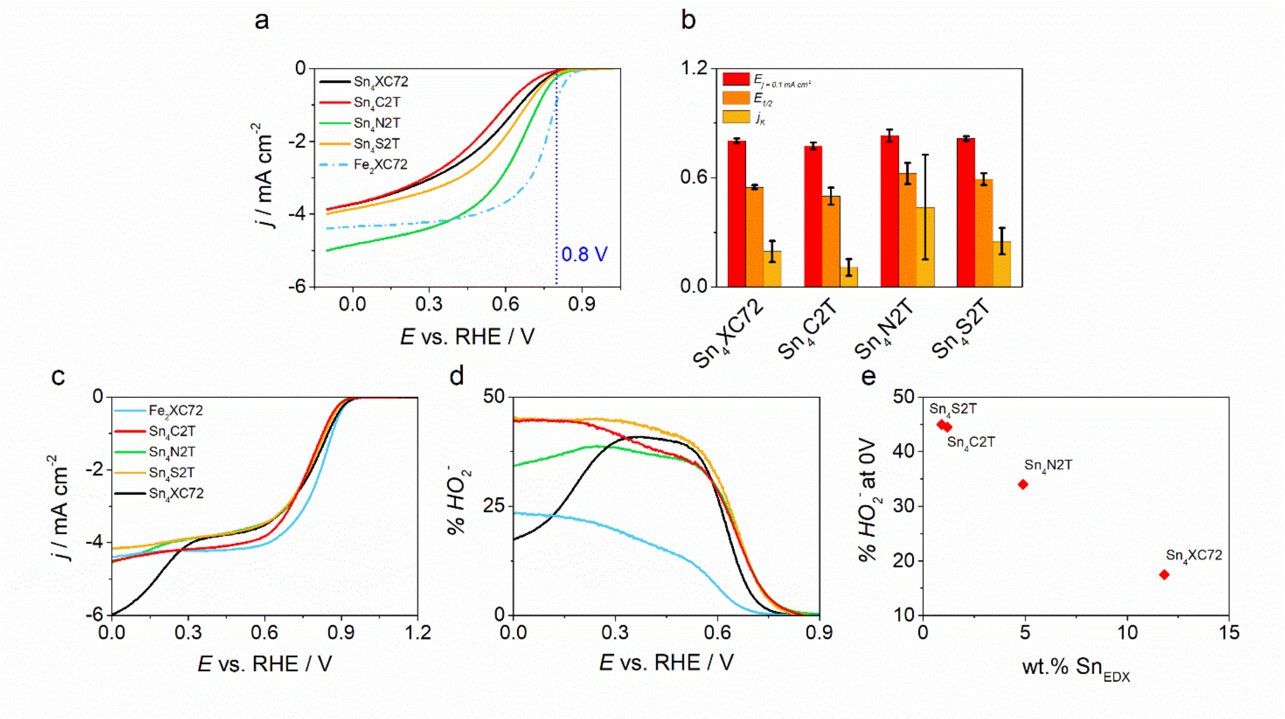

Figure S3. a, b) LSV and activity summary for measurement in acid electrolyte for Sn_4XC72, Sn_4C2T, Sn_4SNT and Sn_4S2T. For the same sample c) LSV, d) peroxide production in alkaline electrolyte and e) variation of peroxide yield as a function of Sn content. Fe_2XC72 was also reported for sake of comparison.

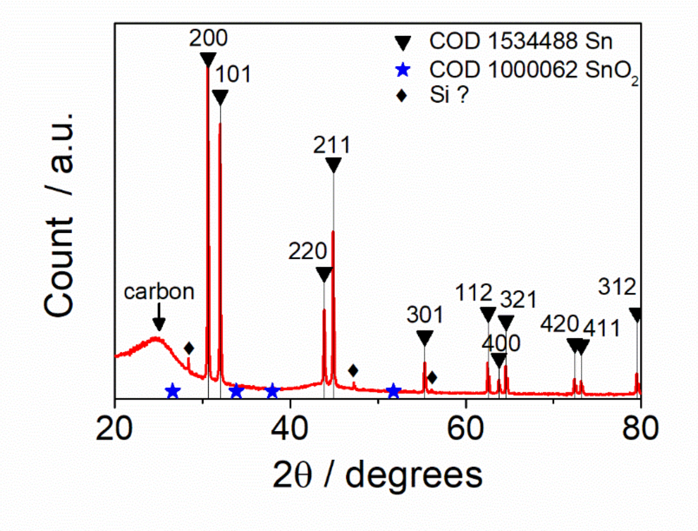

Figure S4. XRD response for Sn_4XC72, diamond shaped labelled peak could be due to the silicon sample holder.

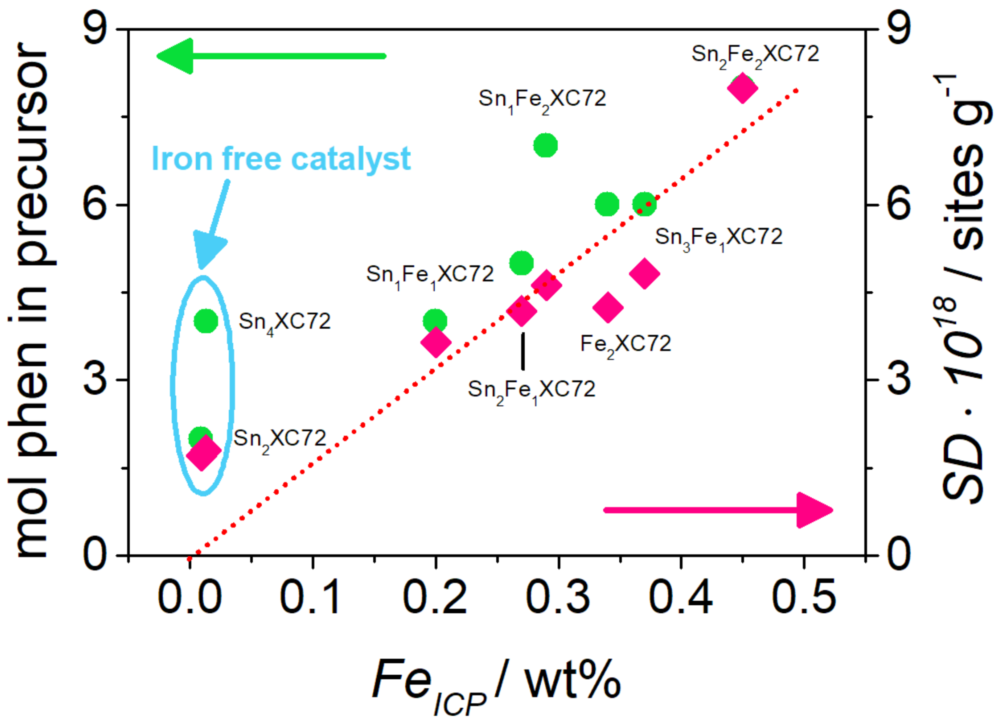

Figure S5. Correlation between phenanthroline in the precursor mixture and SD vs. the amount of iron detected by ICP-MS.

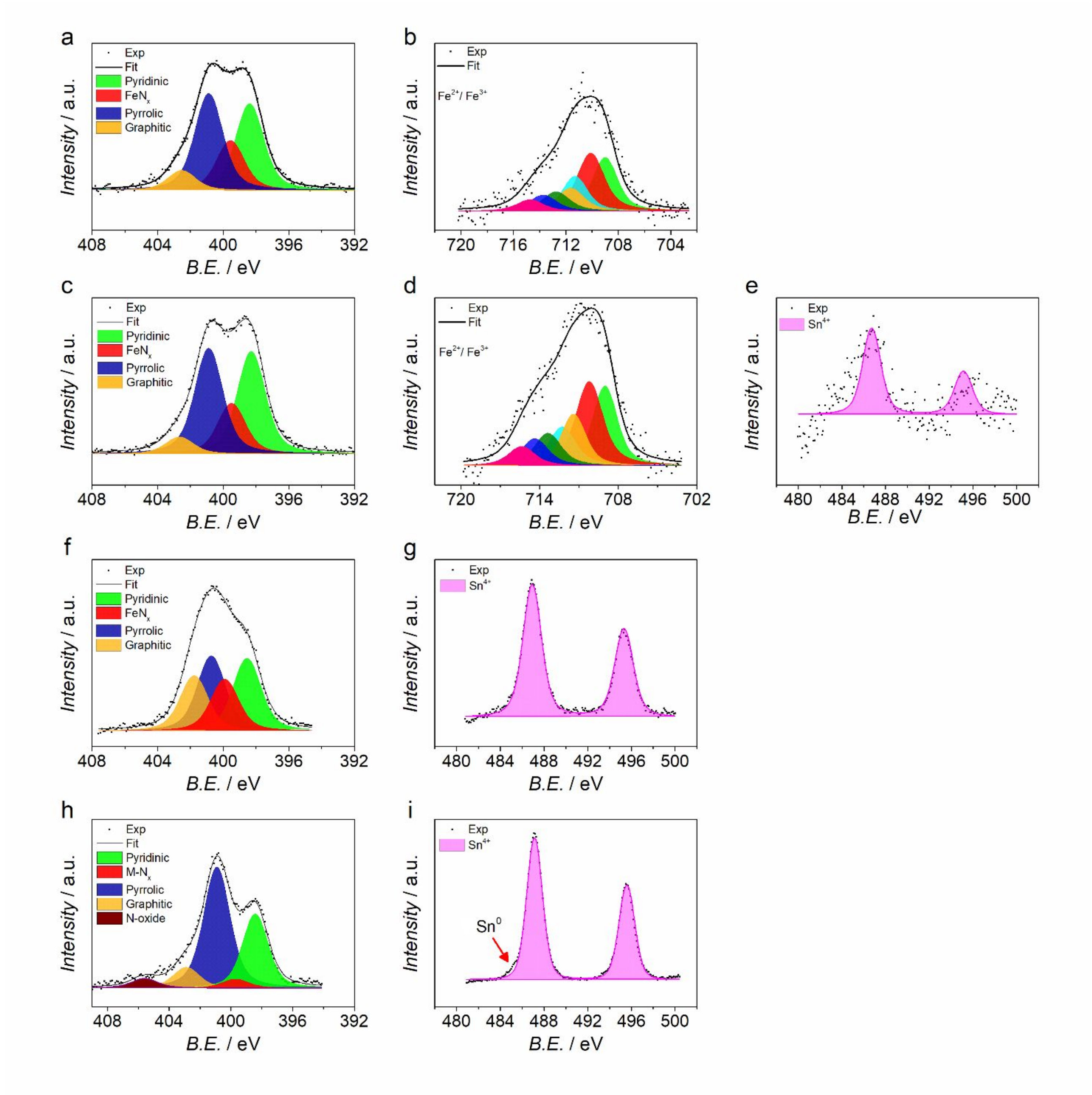

Figure S6: High resolution XPS peaks and deconvolution for (a,b) Fe_2XC72, (c,d,e) Sn_1Fe_2XC72, (f,g) Sn_2Fe_2XC72 and (h,i) Sn_4XC72. a, c, f, h) N 1s, b, d) Fe 2p e, g, i) Sn 3d.

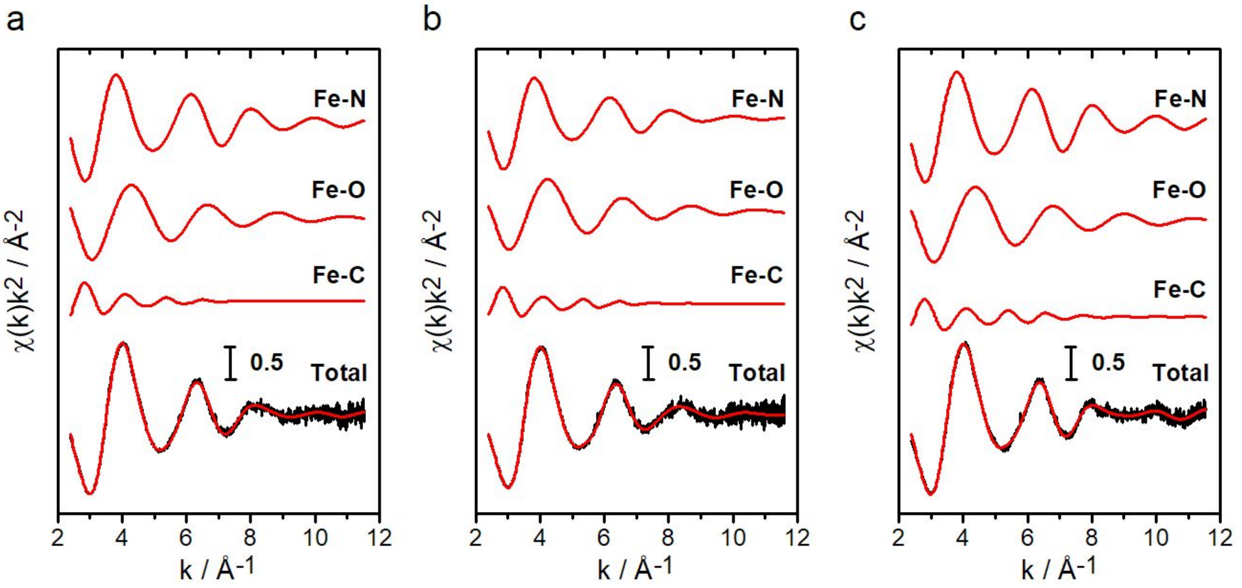

Figure S7. Fe K-edge EXAFS analysis of a) Fe_2XC72, b) Sn_1Fe_2XC72 and c) Sn_2Fe_2XC72 via the GNXAS program. From top to bottom: Fe-N, Fe-O, and Fe-C contributions included in the fit, and the total theoretical signal (red line) superimposed to the experimental one (black line).

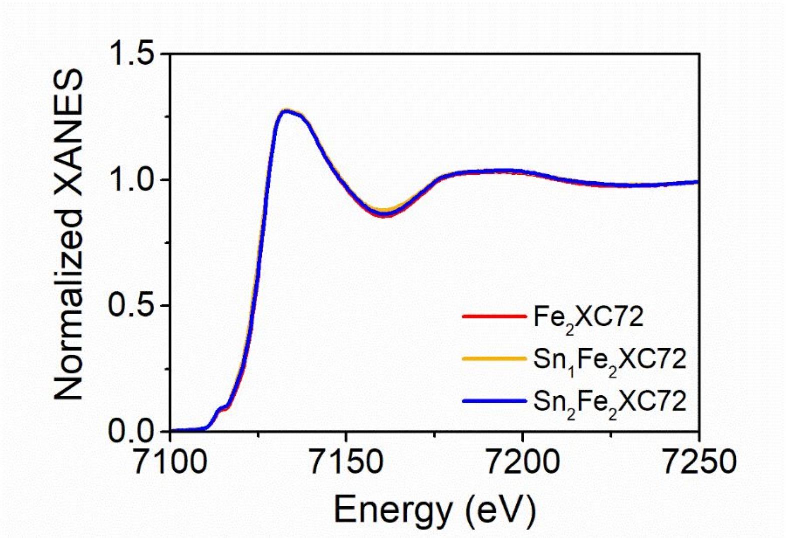

Figure S8. Fe K-edge XANES spectra of Fe_2XC72 (red line), Sn_1Fe_2XC72 (orange line) and Sn_2Fe_2XC72 (blue line).

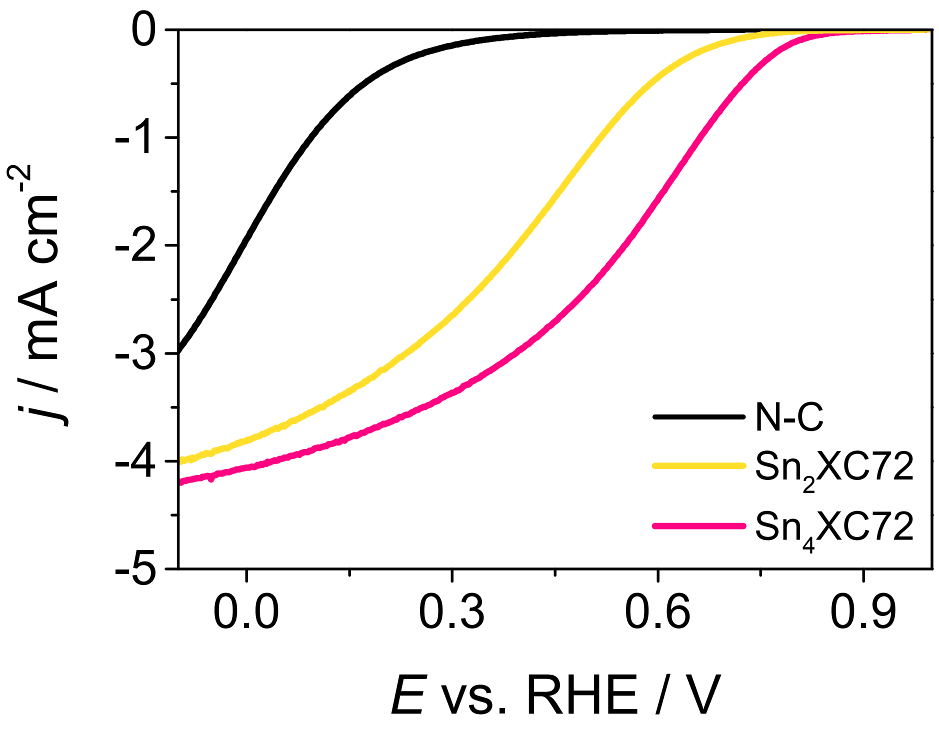

Figure S9. Comparison between Sn_x samples and a metal-free-N-doped carbon in 0.5 M H_2SO_4

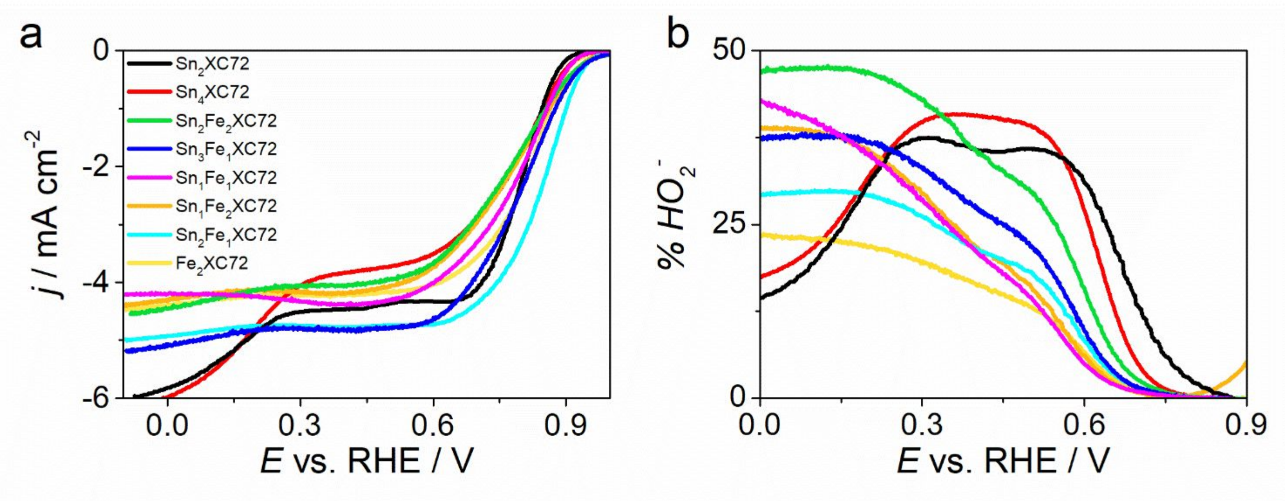

Figure S10. a) LSVs and b) Peroxide yield in 0.1 M KOH

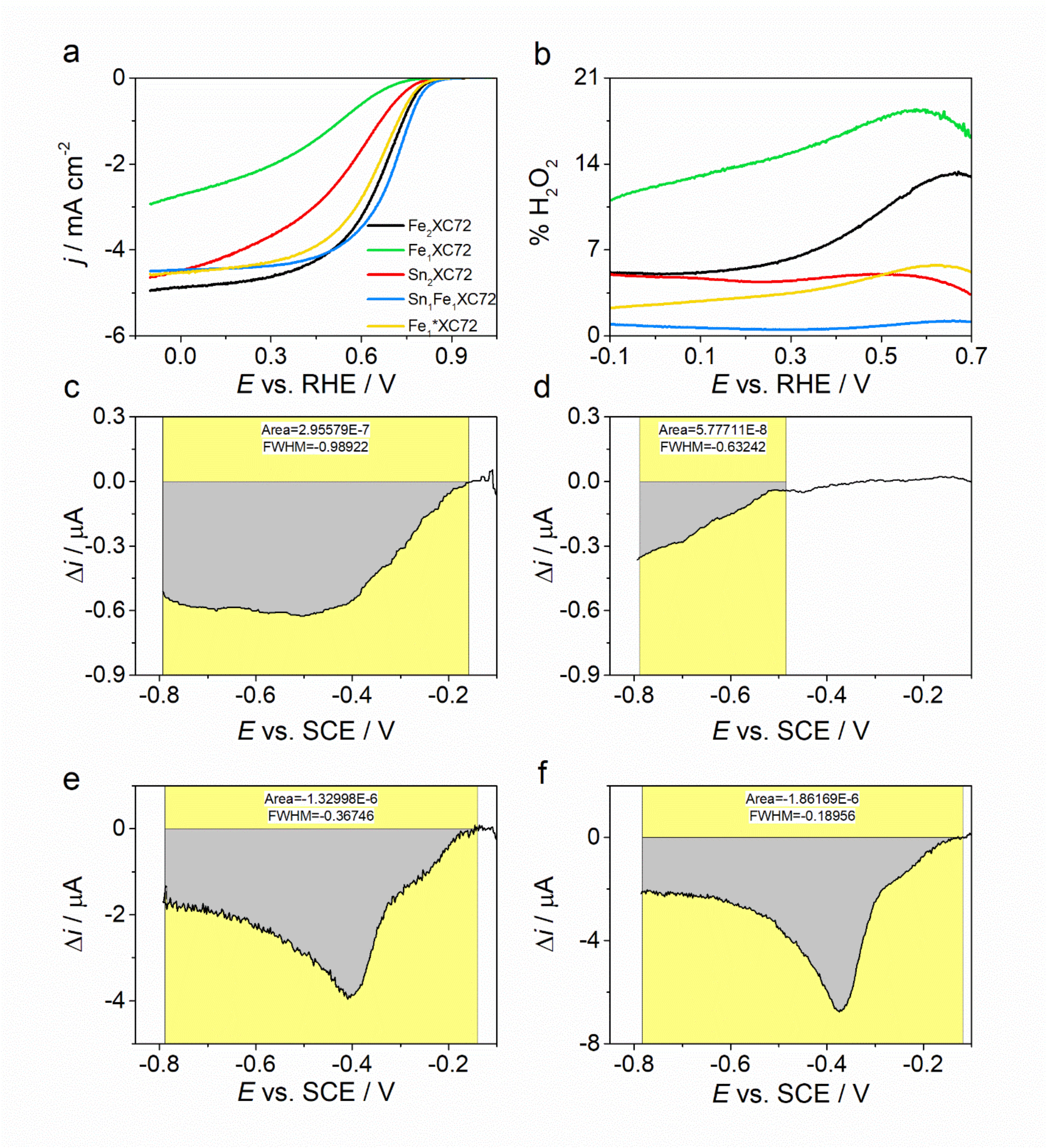

Figure S11. a) Effect of Sn and phenanthroline on activity and b) hydrogen peroxide production for Fe_xXC72; c-f) Stripping for Sn_2XC72, metal-free catalyst (N-C), Fe_2XC72 and Sn_2Fe_1XC72.

Supporting References

[1] F. Luo, A. Roy, L. Silvioli, D.A. Cullen, A. Zitolo, M.T. Sougrati, I.C. Oguz, T. Mineva, D. Teschner, S. Wagner, J. Wen, F. Dionigi, U.I. Kramm, J. Rossmeisl, F. Jaouen, P. Strasser, P-block single-metal-site tin/nitrogen-doped carbon fuel cell cathode catalyst for oxygen reduction reaction, Nat. Mater. (2020) 1–9. https://doi.org/10.1038/s41563-020-0717-5.

[2] A. Filipponi, A. Di Cicco, C.R. Natoli, X-ray-absorption spectroscopy and n-body distribution functions in condensed matter. I. Theory, Phys. Rev. B. 52 (1995) 15122–15134. https://doi.org/10.1103/PhysRevB.52.15122.

[3] A. Filipponi, A. Di Cicco, X-ray-absorption spectroscopy and n-body distribution functions in condensed matter. II. Data analysis and applications, Phys. Rev. B. 52 (1995) 15135–15149. https://doi.org/10.1103/PhysRevB.52.15135.

[4] M. Mazzucato, C. Durante, How Determinant is the Iron Precursor ligand in Fe-N-C Single-Site formation and activity for Oxygen Reduction Reaction?, Electrochim. Acta. 394 (2021) 139105. https://doi.org/10.1016/j.electacta.2021.139105.

[5] M. Inaba, A.W. Jensen, G.W. Sievers, M. Escudero-Escribano, A. Zana, M. Arenz, Benchmarking high surface area electrocatalysts in a gas diffusion electrode: Measurement of oxygen reduction activities under realistic conditions, Energy Environ. Sci. 11 (2018) 988–994. https://doi.org/10.1039/c8ee00019k.

[6] G.W. Sievers, GDE-cell online shop, (n.d.). https://gde-cell.com/ (accessed November 2, 2021).
